# Brain Oscillatory and Hemodynamic Activity in a Bimanual Coordination Task Following Transcranial Alternating Current Stimulation (tACS): A Combined EEG-fNIRS Study

**DOI:** 10.3389/fnbeh.2018.00067

**Published:** 2018-04-18

**Authors:** Alisa Berger, Nils H. Pixa, Fabian Steinberg, Michael Doppelmayr

**Affiliations:** ^1^Department of Sports Psychology, Institute of Sport Science, Johannes Gutenberg-University Mainz, Mainz, Germany; ^2^Centre for Cognitive Neuroscience, Paris Lodron University of Salzburg, Salzburg, Austria

**Keywords:** high-definition tACS, after-effects, alpha oscillations, beta oscillations, Hboxy, bimanual movements

## Abstract

Motor control is associated with synchronized oscillatory activity at alpha (8–12 Hz) and beta (12–30 Hz) frequencies in a cerebello-thalamo-cortical network. Previous studies demonstrated that transcranial alternating current stimulation (tACS) is capable of entraining ongoing oscillatory activity while also modulating motor control. However, the modulatory effects of tACS on both motor control and its underlying electro- and neurophysiological mechanisms remain ambiguous. Thus, the purpose of this study was to contribute to gathering neurophysiological knowledge regarding tACS effects by investigating the after-effects of 10 Hz tACS and 20 Hz tACS at parietal brain areas on bimanual coordination and its concurrent oscillatory and hemodynamic activity. Twenty-four right-handed healthy volunteers (12 females) aged between 18 and 30 (*M* = 22.35 ± 3.62) participated in the study and performed a coordination task requiring bimanual movements. Concurrent to bimanual motor training, participants received either 10 Hz tACS, 20 Hz tACS or a sham stimulation over the parietal cortex (at P3/P4 electrode positions) for 20 min via small gel electrodes (3,14 cm^2^ Ag/AgCl, amperage = 1 mA). Before and three time-points after tACS (immediately, 30 min and 1 day), bimanual coordination performance was assessed. Oscillatory activities were measured by electroencephalography (EEG) and hemodynamic changes were examined using functional near-infrared spectroscopy (fNIRS). Improvements of bimanual coordination performance were not differently between groups, thus, no tACS-specific effect on bimanual coordination performance emerged. However, physiological measures during the task revealed significant increases in parietal alpha activity immediately following 10 Hz tACS and 20 Hz tACS which were accompanied by significant decreases of Hboxy concentration in the right hemispheric motor cortex compared to the sham group. Based on the physiological responses, we conclude that tACS applied at parietal brain areas provoked electrophysiological and hemodynamic changes at brain regions of the motor network which are relevant for bimanual motor behavior. The existence of neurophysiological alterations immediately following tACS, especially in the absence of behavioral effects, are elementary for a profound understanding of the mechanisms underlying tACS. The lack of behavioral modifications strengthens the need for further research on tACS effects on neurophysiology and behavior using combined electrophysiological and neuroimaging methods.

## Introduction

Neurons in the human brain are interconnected and form functionally specialized networks. It is the temporally precise coordinated communication between and within these networks that enables the brain’s complex processing capabilities (Schnitzler and Gross, 2005; Sauseng and Klimesch, 2008; Wach et al., 2013). It is thought that synchronous oscillatory activity represents the basic mechanism for functional communication (Wach et al., 2013; Pollok et al., 2015). The communication underlying motor control is represented in a dynamically functional interaction in a cerebello-thalamo-cortical network with synchronized brain oscillations in the alpha (8–12 Hz) and beta (13–30 Hz) range (Davis et al., 2012; Wach et al., 2013). In complex movements such as bimanual coordination, the oscillatory activity at 20 Hz dominates within and between the left and right hemispheric motor networks (Serrien et al., 2003; Rjosk et al., 2016). Swinnen and Wenderoth (2004) revealed that the respective network formation is dependent on the way in which the movements are internally generated or externally (visually) driven. If movements are internally generated, a basal ganglia-supplementary motor area circuit dominates, and a parietal-premotor pathway exerts control when movements are externally driven (Swinnen and Wenderoth, 2004). In the latter case, the posterior parietal cortex (PPC) integrates multi-sensory signals and is responsible for the spatial-temporal coordination of visually controlled movements. The premotor cortex (PMC) prepares and plans movements, whereas, the primary motor cortex (M1) controls the execution and is relevant for motor consolidation. If a given planned movement differs from the executed movements, the cerebellum and the PPC generate error signals to regulate movement impulses (Della-Maggiore et al., 2004). The corpus callosum plays a pivotal role in interhemispheric interaction in bimanually coordinated movements. By connecting the left and right hemispheres it enables the transfer of information and the maintenance of independent processing of one hemisphere (Takeuchi et al., 2012). The electro- and neurophysiological mechanisms underlying bimanual coordination are still controversially debated and far from being fully understood. Evidence exists supporting the functional role of interhemispheric synchronization in the organization of bimanual movements since bimanual movement disorders are accompanied by pathological oscillatory activity, reduced functional coupling of primary sensorimotor regions (Serrien and Brown, 2002) and altered interregional brain synchronization in patients suffering from neurological diseases (Stam et al., 2003; Schnitzler and Gross, 2005; Uhlhaas and Singer, 2006). Nevertheless, unambiguous evidence is missing, thereby preventing the suggestion that altered oscillatory activity is associated with movement disorders. One possibility to investigate the causality between brain oscillations and behavior is the modulation of oscillatory activity in a frequency-specific manner with transcranial alternating current stimulation (tACS) (Zaehle et al., 2010; Polanía et al., 2012). By applying a weak current in the form of sinusoidal waves, tACS is a promising tool to investigate causal relationships between synchronized oscillations, brain activity and cognitive/motor functions.

Numerous studies investigated the after-effects of tACS on perception (Strüber et al., 2014), cognitive functions (Vosskuhl et al., 2015a; Chander et al., 2016; Kasten and Herrmann, 2017), multitasking (Hsu et al., 2017) or motor control and learning (Pogosyan et al., 2009; Pollok et al., 2015; Cappon et al., 2016; Krause et al., 2016; Leunissen et al., 2017). Regarding tACS effects on motor learning, studies demonstrated heterogeneous results because of different system parameters, various motor tasks and high variabilities in tACS responders and non-responders. Whereas improvements in motor learning were demonstrated at 10 Hz tACS (Antal et al., 2008) and 20 Hz tACS (Krause et al., 2016), Pogosyan et al. (2009) reported that 20 Hz tACS slows voluntary movements during a bimanual tracking task (Pogosyan et al., 2009). Investigating tACS effects on interhemispheric interaction, Heise et al. (2017) applied a bilateral tACS montage on the motor cortex concurrently to a bimanual coordination task. While beta synchronization reduced bimanual coordination performance, alpha synchronization promotion was associated with behavioral improvements (Heise et al., 2017).

Concurrent to behavioral studies, additional and important physiological investigations rely mostly on resting-state measurements after tACS (Kasten and Herrmann, 2017). Zaehle et al. (2010) elevated EEG alpha power with tACS at the individual alpha frequency (IAF) and reported a direct electrophysiological evidence for the feasibility of tACS to modulate ongoing oscillatory activity in the human cortex (Zaehle et al., 2010). Others observed enhanced alpha power after 20 min of tACS at the IAF which lasted 90 min (Kasten et al., 2016). The difference between the stimulation group and the sham group was found to be diminished after 70 min due to a natural alpha increase of the sham group (Kasten et al., 2016) indicating that alpha-tACS can enhance the amplitude of the IAF. However, it remains unclear, which physiological mechanisms underlying tACS are responsible for the after-effects, how long potential after-effects may last and whether the effects can be elicited only during rest or also during complex motor behavior.

One approach explaining the mechanisms of tACS is that stimulation impacts intrinsic oscillatory brain activity through entrainment. Entraining a brain oscillation implies that phase and frequency of brain oscillations are modulated to follow the external stimulation (Antal and Paulus, 2013; Herrmann et al., 2013; Helfrich et al., 2014; Vosskuhl et al., 2015b; Ruhnau et al., 2016). Whether a neural oscillator follows the external induced tACS frequency depends on the frequency range around its intrinsic frequency. The closer the intrinsic frequency range of the neural oscillator is to the applied tACS frequency or the higher the intensity of tACS is, then the higher the probability of entrainment. Additionally, many other ratios between stimulation frequency and frequency of the neural oscillator (e.g., 1:2, 1:1, 2:1) are thought to cause an entrainment (Herrmann and Strüber, 2017). If altered oscillatory brain activity remains for some time after stimulation, plastic-related changes evoked through spike-timing dependent plasticity (Feldman, 2012) are believed to be the underlying mechanisms responsible for the after-effects (Zaehle et al., 2010; Polanía et al., 2012; Vossen et al., 2015).

However, the exact mechanism of tACS induced after-effects remains controversial due to varying stimulation parameters across the studies such as duration, intensity, frequency and electrode montage and the fragmentary knowledge regarding the underlying neurophysiological mechanisms of tACS. Therefore, we argue that combining EEG and fNIRS (Chen et al., 2015) to examine tACS is essential and fundamental for the understanding of its effects and clinical implementation as it might allow a multidimensional perspective on basic neural mechanisms (Bergmann et al., 2016; Choe et al., 2016).

Several EEG/fMRI studies revealed how the manipulated oscillatory activity of tACS is represented in the brain’s metabolism while demonstrating that increased alpha and beta amplitudes correlate with deactivation in occipital areas measured by reduced blood oxygenation level dependent (BOLD) contrasts (Cabral-Calderin et al., 2016; Williams et al., 2017). Moreover, Vosskuhl et al. (2016) confirmed that entrainment of EEG alpha oscillations by tACS at the IAF reduces the BOLD response to visual stimuli (Vosskuhl et al., 2016). Cabral-Calderin et al. (2016) observed the strongest effects on BOLD activity following alpha/beta-tACS, but also specified that tACS affects the BOLD signal in a frequency and task-dependent manner. Furthermore, they emphasize that the strongest effects of tACS might exist in regions not necessarily situated below the stimulation electrodes (Cabral-Calderin et al., 2016). In two further neuroimaging studies, an application with fNIRS and transcranial direct stimulation (tDCS) was used (McKendrick et al., 2015; Muthalib et al., 2017), but to our knowledge, combined electro-/neurophysiological investigations of transcranial electrical stimulation (tES) during complex motor behaviors are rare. Only one approach applied simultaneously EEG and fNIRS to a complex bimanual motor task after tDCS (Choe et al., 2016). To investigate the modulatory effects of tDCS on neural functions and bimanual motor learning, 32 participants received either sham or anodal tDCS to the dorsolateral prefrontal cortex (DLPFC) or the left motor cortex (M1) during pilot training in four consecutive daily sessions. Results from days 1 to 4 demonstrated for M1 stimulated subjects that increased parietal alpha power correlated strongly with reduced fNIRS beta-values in M1 channels (Choe et al., 2016). A simultaneous EEG/fNIRS measure of tACS effects on complex motor performance was not carried out prior to this experimentation even though simultaneous recordings of EEG and neuroimaging that directly reflect the tACS effects are the keys for a better understanding of how tACS impacts brain activity (Herrmann and Strüber, 2017).

To summarize, previous studies investigated the effects of tACS on motor processes and brain oscillations. However, the functional role of synchronized oscillatory activity in interhemispheric communication during bimanual coordination, their modulation by tACS and the underlying neurophysiological mechanisms remain ambiguous. Therefore, further research concerning the mechanisms of tACS and its modulatory effect on complex movements is required giving the fact that such knowledge is of clinical relevance for the rehabilitation of bimanual coordination.

Consequently, the central purpose of this study was to investigate the modulatory effects of parietal 10 Hz tACS and 20 Hz tACS on brain oscillations, hemodynamic changes and bimanual coordination using a combined tACS/EEG/fNIRS approach. Assuming that alpha and beta frequencies represent different functions and that tACS provides the possibility to interact with oscillatory activity, we hypothesized that tACS after-effects vary in a frequency dependent manner. Owing to previous work, we hypothesized that parietal 10 Hz tACS evoke synchronized oscillatory activity in the alpha range that lead to reduced interhemispheric interaction and deterioration in bimanual coordination performance (Serrien and Brown, 2002) whereas 20 Hz tACS would promote the natural beta oscillation of bimanual coordination (Rjosk et al., 2016) that leads to an enhanced performance.

## Materials and Methods

The study was performed in accordance with the latest revision of the Declaration of Helsinki. Experimental procedures were performed along the recommendations of the Deutsche Gesellschaft für Psychologie (DGPs) and approved by the local ethics committee of the Johannes Gutenberg-Universität Mainz. Participants were informed about all relevant issues of the study and gave their written informed consent prior to the initiation of the experiment.

### Participants

Twenty-four healthy subjects (12 females) aged between 18 and 30 (*M* = 22.35; *SD* = 3.62) were recruited to participate in this double-blind study. All participants were right-handed according to the Edinburg handedness-scale (Oldfield, 1971), without any neurological or psychological disorders and with normal or corrected-to-normal vision. All participants were requested to disclose pre-existing neurological and psychological conditions, medical conditions, drug intake and alcohol or caffeine intake during the past week. Prior to initiating the study, participants were randomly assigned to a group receiving either 10 Hz tACS, 20 Hz tACS or sham stimulation (see **Table 1**).

**Table 1:** Subject characteristics by group.

	Group		
Characteristics	Sham	10 Hz tACS	20 Hz tACS
Sex (female/male)	(5/3)	(2/6)	(5/3)
Age (*M* ±*SD*)	23 ± 3	22 ± 2	22 ± 3

### Bimanual Coordination Task

**FIGURE 1:**
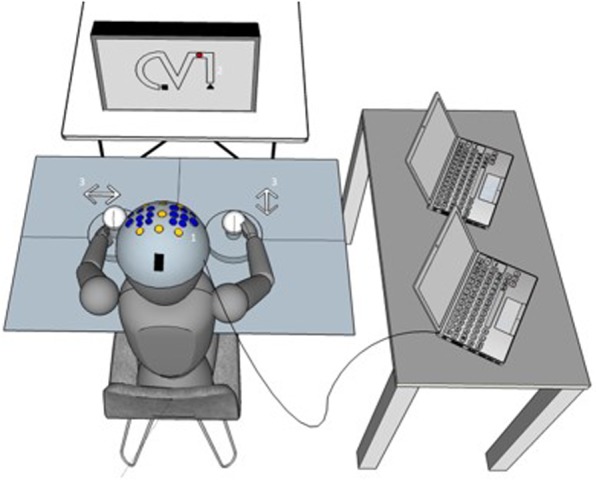
Experimental setup. (1) The tACS and neuroimaging (EEG/fNIRS) setup is shown on a virtual subject. (2) The bimanual coordination task. In each trial, participants were instructed to operate the two joysticks (3) in order to direct a virtual dot on the screen as fast and as accurate as possible from the starting point (Δ) to the endpoint (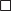) of a predefined route. More details regarding the coordination task are in the text (paragraph Bimanual coordination task in section “Materials and Methods”).

The so-called 2HAND task is a well-known psychological diagnostic tool used in the examination of bimanual coordination and eye-hand coordination (Vienna Test System, Schuhfried, Austria). As depicted in **Figure 1**, it consists of a monitor, a keyboard, and two joysticks with fixed steering plates. This fixed steering plate restricts the participant’s control of the left and right joysticks in a horizontal and a vertical direction, respectively. The participants were instructed to operate the two joysticks in order to direct a virtual dot as fast and as accurately as possible from the starting point (Δ) to the endpoint (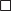) of a predefined route which is presented on the screen while trying to avoid making mistakes. That is, a mistake is defined as the leaving of the shape (see **Figure 1**). This task requires the subject to guide the dot as smoothly as possible along the shape, which necessitates precisely timed and visually guided coordination between the left and right joysticks simultaneously.

### tACS/EEG/fNIRS Approach

**FIGURE 2:**
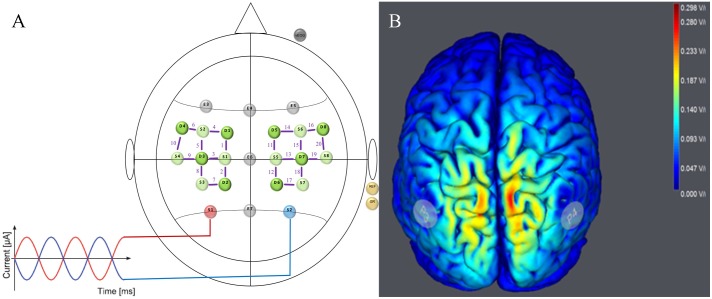
Stimulation and neuroimaging setup. **(A)** tACS/EEG/fNIRS Montage for Parietal Stimulation. HD-tACS electrodes were positioned at P3 (red electrode = stimulation) and P4 (blue electrode = return). EEG was measured from five positions (F3, Fz, F4, Cz, and Pz) following the international 10–10 system (gray electrodes). fNIRS sources (light green) and detectors (dark green) are shown over the left and right motor cortices with channels (Source–Detector) depicted as purple lines (FC3–FC5, FC3–FC1, FC3–C3, C1–FC1, C1–C3, C1–CP1, C5–C3, C5–FC5, CP3–C3, CP3–CP1, FC4–FC2, FC4–FC6, FC4–C4, C2–FC2, C2–C4, C2–CP2, C6–FC6, C6–C4, CP4–CP2, und CP4–C4). **(B)** Simulated Electrical Field. Predicted electrical field intensities with a tACS application on P3/P4 (StartStim, Neuroelectrics, Spain). Full details regarding stimulation and brain activity measures are in the text (paragraph tACS/EEG/fNIRS approach).

To realize the tACS/EEG/fNIRS approach, two devices were combined providing transcranial current stimulation (tCS) and EEG measures in one device (StarStim, Neuroelectrics, Spain) and a 20-channel fNIRS device (NIRSport, nirx, Germany) (see **Figure 2A**). For tACS and EEG, small, round gel electrodes (Ag/AgCl, 3.14 cm^2^) were used (Pi-electrodes, Neuroelectrics) and realized a so-called high-definition tACS (HD-tACS). Eight electrodes were positioned in a non-conductive neoprene cap following the international 10–10 EEG system (Jurcak et al., 2007). Following the calculation of the electrical field of HD-tACS, electrode positions were selected based on the computational model by Ruffini et al. (2014). Based on previous studies which elicited and demonstrated physiological after-effects of IAF tACS in alpha oscillations during rest (Zaehle et al., 2010; Vossen et al., 2015), the stimulation electrodes were placed at P3 (stimulation electrode) and P4 (return electrode) to cover the parietal cortices (see **Figure 2B**). These cortices play a functional role in visually guided bimanual coordination (Swinnen and Wenderoth, 2004; Buneo and Andersen, 2006; Culham et al., 2006).

The alternating current was delivered at an intensity of 1 mA resulting in a current density of 0.32 mA/cm^2^ in the skin under the stimulation electrodes. The stimulation groups received 20 min of tACS either at 10 Hz or 20 Hz with 15 seconds (s) ramp-up and ramp-down at the beginning and at the end of stimulation. The control group (sham) received only 60 s of tACS (including 15 s ramp-up and ramp-down) to induce the physical sensation associated with tACS (e.g., tingling or itching). Thereby, the impedance values are limited to 10 kΩ during tACS and impedance values were controlled throughout the duration of the whole experiment. To ensure that participants and the investigator are blind to the stimulation condition, the password-protected double-blind mode of the control software (StarStim, Instrument Controller v 1.4, Neuroelectrics, Spain) was used. Further five electrodes were positioned at F3, Fz, F4, Cz, and Pz and resulted in the provision of EEG measurements also during tACS. Before and after tACS, the actual stimulation electrodes (P3 and P4) were also used for EEG, resulting in seven EEG-channels before and after tACS and five EEG-channels during tACS. Additionally, a vertical electro-oculogram (EOG) was recorded from an electrode over the right eye. The common mode sense (CMS) and driven right leg (DRL) connections were placed on the right mastoid and corresponded to the electrical reference. Overall, the sampling rate was set at 500 Hz.

For fNIRS measurement, a standard configuration for motor tasks was used to accommodate the fNIRS sources and detectors with tACS-EEG-electrodes. 20 channels (source-detector pairs) were positioned at an interval of 3 cm over the left motor cortex (10 channels) and the right motor cortex (10 channels) covering the arm and hand area of both M1 (see **Figure 2A**). fNIRS data were recorded at a sampling rate of 7.81 Hz. Near-infrared light at the wavelengths of 760 and 850 nm which are thought to be optimal for measuring both chromophores (Hboxy/Hbdeoxy) were predefined.

### Debriefing

After completing the experiment, participants rated their feelings and their state of arousal. They filled out a translated version of an adverse effects questionnaire where they had to rate the type and intensity of adverse effects including factors such as tiredness, concentration level, cognitive and motor enhancement, pains (1 – none, 5 – moderate, 9 – severe) and how much they were related to the stimulation (1 – none, 5 – uncertain, 9 – definite). In addition, they were requested to guess their experimental condition to ensure that they were naive toward real or sham stimulation. Therefore, a questionnaire asked whether they believe to have received real or sham stimulation or whether they are not sure. A chi-square test was used to assess whether subjects were able to correctly identify stimulation or sham.

### Experimental Design and Procedure

**FIGURE 3:**
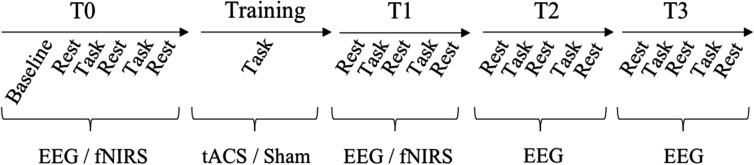
Experimental design. Upper line depicts the timeline of test and training sessions. During the test session, participants performed two blocks of the bimanual coordination task (five trials each) and three blocks of rest with eyes closed and eyes open before tACS (T0) and three times after tACS (immediately = T1, 30 min = T2, 1 day = T3). During tACS/training (20 min), participants performed three blocks with ten trials of the bimanual coordination task each and resting states in between. Lower line presents when tACS, EEG and fNIRS were recorded.

A pre-/post-test design with retention-tests was utilized to investigate tACS effects on online (within day) and offline motor learning (across days) (Wessel et al., 2015) as well as its modulatory effects which were measured by EEG and fNIRS (see **Figure 3**). At the beginning of the 1st day, participants completed all forms and received a standardized written instruction along a video-based demonstration of the required bimanual coordination task. To familiarize participants with the equipment and the bimanual coordination task, an acquisition phase with two practice trials followed.

Before tACS (T0) and at three time-points after tACS (immediately = T1, 30 min = T2, 1 day = T3), participants performed trials of the bimanual coordination task and resting state measurements. The bimanual coordination task contained two blocks with five trials. Before, between and after these two bimanual coordination blocks, resting state measurements with eyes closed and eyes open were performed for 1 min each (see **Figure 3**). Following T0, training started with thirty trials of the bimanual coordination task which lasted 20 min. Concurrent to this training, participants received either 10 Hz tACS, 20 Hz tACS or sham stimulation.

Whereas electrophysiological EEG data were collected at all points of time, fNIRS was only used at T0, training and T1, thereby, focusing the effects immediately after tACS while avoiding well-known side-effects of fNIRS (e.g., pressure pain on the head). Before the experiment started, resting state brain activity were collected for 30 s and served as baseline for NIRS analyses. In the following, groups are referred to as (1) 10 Hz tACS, (2) 20 Hz tACS and (3) sham.

### Data Analyses

Statistical analyses were performed using IBM SPSS 23. All variables were normally distributed as assessed by the Shapiro–Wilk test (*p* > 0.05). Examining statistical requirements, Levene’s test was used to check for homogeneity and Mauchly’s test was used to check for a violation of sphericity. Several factorial mixed ANOVAs were calculated to examine learning and tACS related changes in bimanual coordination performance and in alpha/beta activity. The exact definition of factors and factor levels of each ANOVA are specified below. For clarity, the ANOVA factors were presented once again at the respective positions in the result section. The overall level of significance was set at *p* ≤ 0.05. If a violation of sphericity was detected (*p* < 0.05) and a Greenhouse–Geisser epsilon 𝜀 > 0.75 existed, Huynh–Feldt corrected *p*-values are reported. Otherwise (epsilon 𝜀 < 0.75), a Greenhouse–Geisser correction is applied. Effects sizes (Es) of ANOVAs are given in partial eta-squared (${\text{η}}_{\text{p}}^{2}$), where ${\text{η}}_{\text{p}}^{2}$ = 0.01 indicates a small effect, ${\text{η}}_{\text{p}}^{2}$ = 0.06 indicates a medium effect and ${\text{η}}_{\text{p}}^{2}$ = 0.14 indicates a large effect.

### Bimanual Coordination Task

Movement time (s) across 10 trials was calculated once before tACS (baseline = T0), immediately after tACS (=T1), 30 min after tACS (=T2) and 1 day after tACS (=T3). Next, for each task completion the differences from the pre-stimulation baseline were computed for further analysis. A two-way analysis of variance (ANOVA) with repeated measures (mixed-design) for TIME (T0, T1, T2, and T3) and GROUP (10 Hz tACS, 20 Hz tACS, Sham) was conducted to evaluate any performance changes in bimanual coordination over time and between the groups. Bonferroni-adjusted *post hoc* analysis was used to identify significant differences in bimanual coordination performance between days and groups in case of significant effects.

### Electrophysiological Data (EEG)

Electrophysiological data were pre-processed using the Brain Vision Analyzer 2.0 (Brain Products, Gilching, Germany) by applying a 0.5 Hz high-pass and a 50 Hz low-pass filter. Horizontal and vertical eye movements were corrected (Gratton and Coles) and epochs containing further artifacts were visually identified and manually rejected. For the evaluation of sustained changes in alpha activity over time, three different conditions (1) eyes closed, (2) eyes open, and (3) bimanual coordination were subdivided into 1 min blocks in the sessions of task execution before (T0) and after stimulation (T1, T2, and T3). Each block was segmented in 1 s epochs, wherefore, a fast fourier transformation (FFT) was subsequently computed. Frequency spectra of the artifact-free segments were averaged for each block and the mean activity (μV) in the alpha range (8–12 Hz) and beta range (18–22 Hz) was calculated and exported. For statistical comparisons, changes to T0 (μV) in alpha and beta activity were analyzed for eyes closed, eyes open and task and were fed separately into different ANOVAs. Firstly, a three-way repeated measure ANOVA with the within factors TIME (T0, T1, T2, and T3) and LOCATION (P3, P4, F3, Fz, F4, Cz, and Pz) and the between factor GROUP (10 Hz tACS, 20 Hz tACS, Sham) was calculated to check for global after-effects of tACS. Secondly, a two-way repeated measure ANOVA with the within factor TIME (T0, T1, T2, and T3) and the between factor GROUP (10 Hz tACS, 20 Hz tACS, Sham) was calculated for the stimulation electrodes P3 and P4 to check location-specific after-effects of tACS in the stimulated area. Changes in alpha and beta activity over time (pre to post tACS) or differences between groups were evaluated by using the Bonferroni-corrected *t*-tests separately for the resting states and the bimanual coordination task. Furthermore, in order to visually demonstrate statistical significant effects, frequency spectra (1–30 Hz) of P3 and P4 and alpha/beta topographies for all conditions and groups were presented in addition to the activity changes over time. Electrophysiological data collected during tACS were not further analyzed due to large tACS induced artifacts.

### Brain Oxygenation (fNIRS)

Brain oxygenation raw data were preprocessed and analyzed within the MATLAB-based nirsLAB analysis package (Nirx Medical Technologies, Glen Head, NY, United States, “Biomedical Optics”). Concentration changes of Hboxy, Hbdeoxy, and Hbtot were (1) corrected for discontinuities, steps and spikes and (2) band-pass filtered from 0.01 to 0.2 Hz to remove slow drifts in the signal related to respiratory or cardiac rhythm. For calculating the hemodynamic response function based on the modified Beer-Lambert law, the distance between the source-detector pairs were computed within nirsLAB in accordance with the montage of the headcap.

Then, 30 s of the resting states (eyes closed/eyes open) and one min from each bimanual coordination block were used to specify the segment eyes closed, eyes open and task. The averaged baseline concentration values were subtracted from these rest-evoked and task-evoked concentration measurements. The averaged concentration value of ΔHboxy was computed separately for each subject, time (T0/T1), channel, resting state (eyes closed/eyes open) and task. Statistical analyses focused on the increases in ΔHboxy, because these appear to reflect task-related cortical activation more directly than decreases in ΔHbdeoxy as evidenced by the stronger correlation between the former and the BOLD signal measured by fMRI (Strangman et al., 2002). To compute statistical significances of group-averaged Hboxy concentration changes (ΔHboxy) caused by tACS, Statistical Parametric Mapping (SPM) was applied. SPM values were further used to estimate beta-values for the subtraction of ΔHboxy before tACS from ΔHboxy after tACS (SPM, nirsLAB, v2016.05). For each participant, a *t*-statistic-threshold (*p* < 0.05) beta-image was computed for baseline-subtracted rest- or task-evoked ΔHboxy before (T0) and immediately after tACS (T1). For group analysis, t-statistic maps (t-contrast: difference between two groups’ hemodynamic responses to T0 and T1) were generated from the averaged beta-values. If the *t*-statistic exceeded the *p*-value threshold of 0.05 (*t*-value < -2.4 and >2.4), the concentration values were determined to be significant and marked colored in the respective map. Furthermore, time courses of ΔHboxy concentrations were calculated for all conditions, groups and channels.

## Results

### Resting Alpha Activity (8–12 Hz) With Eyes Closed

#### Global Effect of tACS

To analyze the global after-effect of tACS on alpha activity, the three-way rmANOVA with all EEG positions revealed a significant main effect for GROUP [*F*(2,21) = 3.466, *p* < 0.05, ${\text{η}}_{\text{p}}^{2}$ = 0.25], a significant main effect for TIME [*F*(3,63) = 3.665, *p* < 0.05, ${\text{η}}_{\text{p}}^{2}$ = 0.15], a significant main effect for LOCATION [*F*(4,91) = 4.769, *p* < 0.01, ${\text{η}}_{\text{p}}^{2}$ = 0.19] and a significant TIME × LOCATION interaction [*F*(9,181) = 2.353, *p* < 0.05, ${\text{η}}_{\text{p}}^{2}$ = 0.10]. The TIME × LOCATION interaction demonstrates that there are significant differences between alpha activity on the eight EEG positions and the four time-points, independently of the GROUP. Neither TIME × GROUP interaction [*F*(6,63) = 1.250, *p* = 0.29, ${\text{η}}_{\text{p}}^{2}$ = 0.11] nor LOCATION × GROUP interaction [*F*(9,90) = 1.120, *p* = 0.35, ${\text{η}}_{\text{p}}^{2}$ = 0.11] nor the three-way interaction TIME × LOCATION × GROUP [*F*(17,181) = 0.774, *p* = 0.83, ${\text{η}}_{\text{p}}^{2}$ = 0.07] revealed significance. The topographies in **Figure 6A** show changes in alpha activity over time for the groups Sham, 10 Hz tACS and 20 Hz tACS.

#### Location-Specific Effect of tACS

**FIGURE 4:**
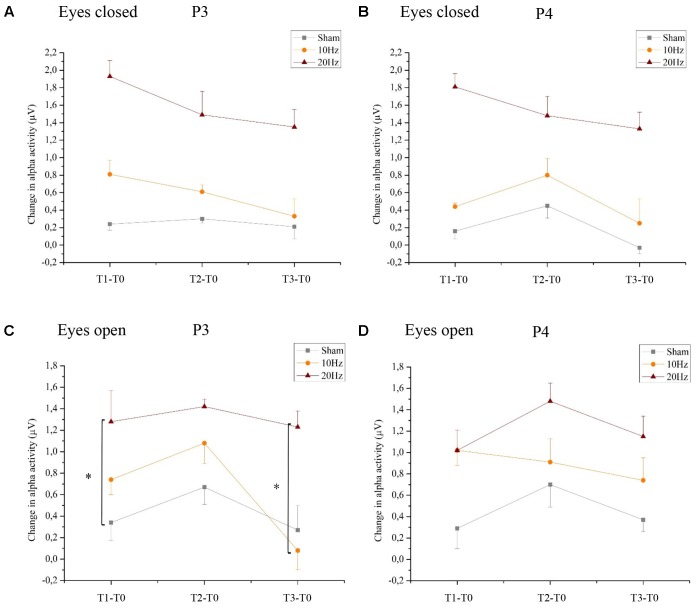
Changes in alpha activity in rest. Changes in alpha activity (μV) in rest from the interval preceding tACS (T0) to T1 (immediately after tACS), T2 (30 min after tACS) and T3 (1 day after tACS) for Sham (gray), 10 Hz tACS (orange) and 20 Hz tACS (ruby). Depicted are means and standard deviations. Note that difference scores are depicted and statistical analysis revealed a significant TIME and GROUP^∗^TIME interaction effect. The main effects as well as the interaction effects are due to significant changes from T0. All statistical details are depicted in the text [see paragraph Resting alpha activity (8–12 Hz) with eyes closed/open in section “Results”). Significantly different changes in alpha activity after tACS to T0 between the groups are marked and depicted with asterisks (^∗^ depicts *p* < 0.05). **(A)** eyes closed P3, **(B)** eyes closed P4, **(C)** eyes open P3, **(D)** eyes open P4.

**FIGURE 5:**
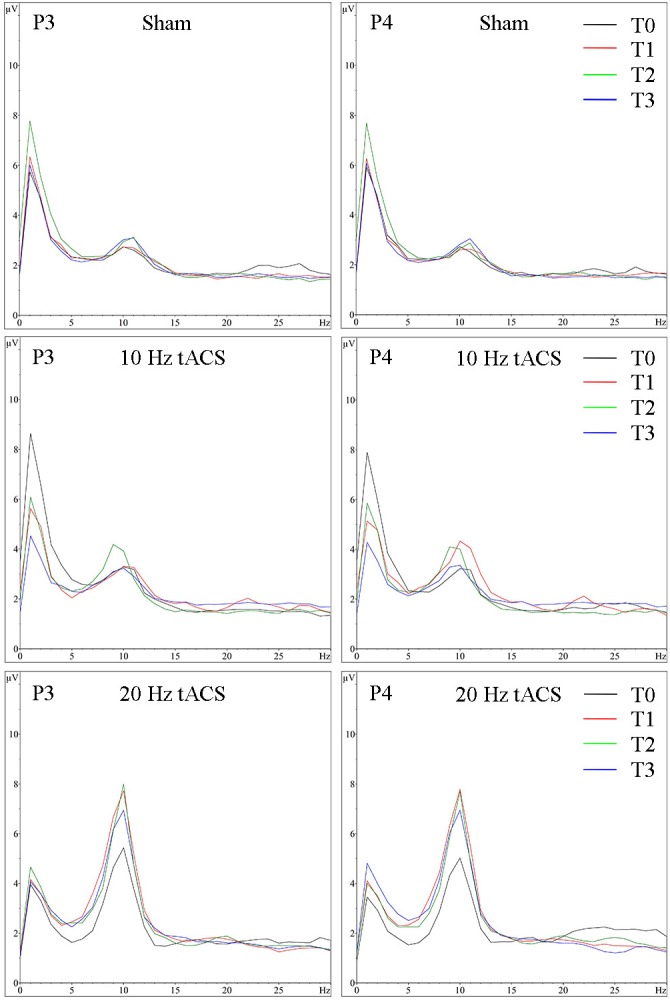
Group averaged EEG activity in rest with eyes closed. Frequency spectrum (amplitude in μV) on P3 **(left)** and P4 **(right)** for the interval with eyes closed preceding tACS (T0 = black), immediately after tACS (T1 = red), 30 min after tACS (T2 = green) and 1 day after tACS (T3 = blue) for Sham (at the top), 10 Hz tACS (in the middle), 20 Hz tACS (at the bottom).

Analyzing the location-specific effects of tACS on alpha oscillatory activity in the stimulated parietal area, an ANOVA was computed for P3 and P4 separately. The two-way rmANOVA for P3 demonstrated a significant main effect for TIME [*F*(3,63) = 5.470, *p* < 0.05, ${\text{η}}_{\text{p}}^{2}$ = 0.22] and a significant main effect for GROUP [*F*(2,21) = 3.125, *p* < 0.05, ${\text{η}}_{\text{p}}^{2}$ = 0.23]. The interaction TIME × GROUP did not reach significance [*F*(6,63) = 1.358, *p* = 0.24, ${\text{η}}_{\text{p}}^{2}$ = 0.12] (see **Figure 4A**). The two-way rmANOVA for P4 showed a significant main effect for TIME [*F*(3,63) = 4.209, *p* < 0.05, ${\text{η}}_{\text{p}}^{2}$ = 0.17] and a significant main effect for GROUP [*F*(2,21) = 3.361, *p* < 0.05, ${\text{η}}_{\text{p}}^{2}$ = 0.24] (see **Figure 4B**). The interaction TIME x GROUP did not reach significance [*F*(6,61) = 1.259, *p* = 0.29, ${\text{η}}_{\text{p}}^{2}$ = 0.11]. Frequency spectra of P3 and P4 for the groups Sham, 10 Hz tACS and 20 Hz tACS are presented in **Figure 5**.

### Resting Beta Activity (18–22 Hz) With Eyes Closed

**FIGURE 6:**
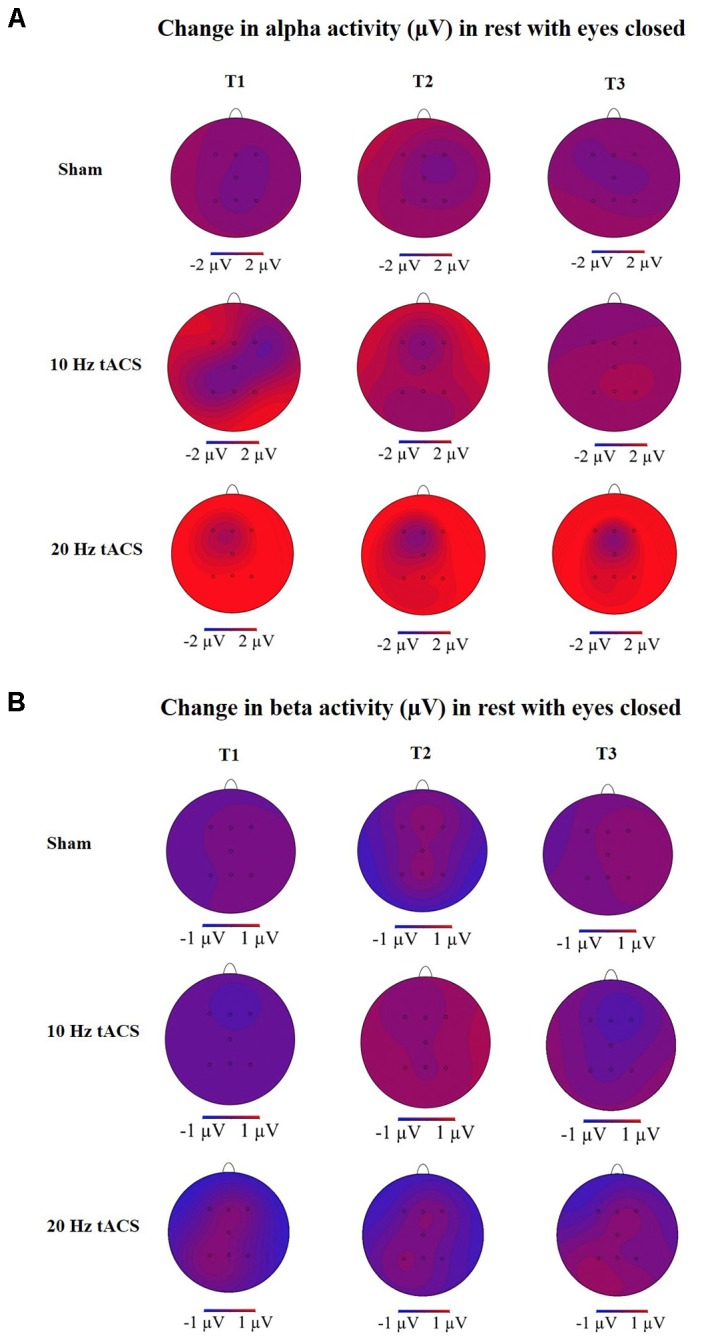
Topographies of group averaged alpha and beta activity changes. **(A)** Changes in alpha activity (μV) in rest from the interval with eyes closed preceding tACS (T0) to T1 (immediately after tACS), T2 (30 min after tACS), and T3 (1 day after tACS) for Sham (at the top), 10 Hz tACS (in the middle), 20 Hz tACS (at the bottom). **(B)** Changes in beta activity (μV) in rest from the interval with eyes closed preceding tACS to T1 (immediately after tACS), T2 (30 min after tACS) and T3 (1 day after tACS) for Sham (at the top), 10 Hz tACS (in the middle), 20 Hz tACS (at the bottom). Note that these topographies are based on seven electrode positions only.

Statistical analysis of beta-band activity (18–22 Hz) using a three-way rmANOVA with the factors TIME, LOCATION, and GROUP revealed neither significant main effects nor significant interactions. As beta-band was completely unaffected by the stimulation protocol, those values will not be further visually or statistically reported. The topographies for beta activity are presented in **Figure 6B**, which additionally demonstrate that the beta-band was completely unaffected by the stimulation protocol.

### Resting fNIRS Activity (ΔHboxy) With Eyes Closed

The fNIRS results demonstrated significant decreases in Hboxy concentration on the left and right hemisphere (*p* < 0.05) before and after training/tACS (see Supplementary Tables S1A,B). According to that, resting state with eyes closed is accompanied by decreased oxygenated hemoglobin in channels covering areas relevant for motor behavior. In addition, Hboxy *t*-values during EC did not differ significantly from T0 and T1 indicating that ΔHboxy concentrations were not influenced by training or tACS (see Supplementary Tables S1C, S2, S3).

### Resting Alpha Activity (8–12 Hz) With Eyes Open

#### Global Effect of tACS

For all electrodes, the three-way rmANOVA revealed a significant main effect for TIME [*F*(3,63) = 12,443, *p* < 0.01, ${\text{η}}_{\text{p}}^{2}$ = 0.37], a significant main effect for LOCATION [*F*(3,66) = 6,036, *p* < 0.01, ${\text{η}}_{\text{p}}^{2}$ = 0.22] and a significant TIME × LOCATION interaction [*F*(11,235) = 2,829, *p* < 0.05, ${\text{η}}_{\text{p}}^{2}$ = 0.12]. The TIME × LOCATION interaction indicates that significant differences of alpha activity on the eight EEG positions to the four time-points exist, and that these are independent of the stimulation condition. Bonferroni-corrected *post hoc t*-tests revealed a significantly different alpha activity between P4 and F4 immediately after tACS, independent of tACS (*p* < 0.05). This indicates a location-specific response of frontal and parietal areas on the right hemisphere to training. The different alpha activity evolvement over time is visually reflected in the topographies for the three groups (see **Figure 8A**) by demonstrating stronger alpha activity changes in parietal areas compared to frontal areas.

#### Location-Specific Effect of tACS

**FIGURE 7:**
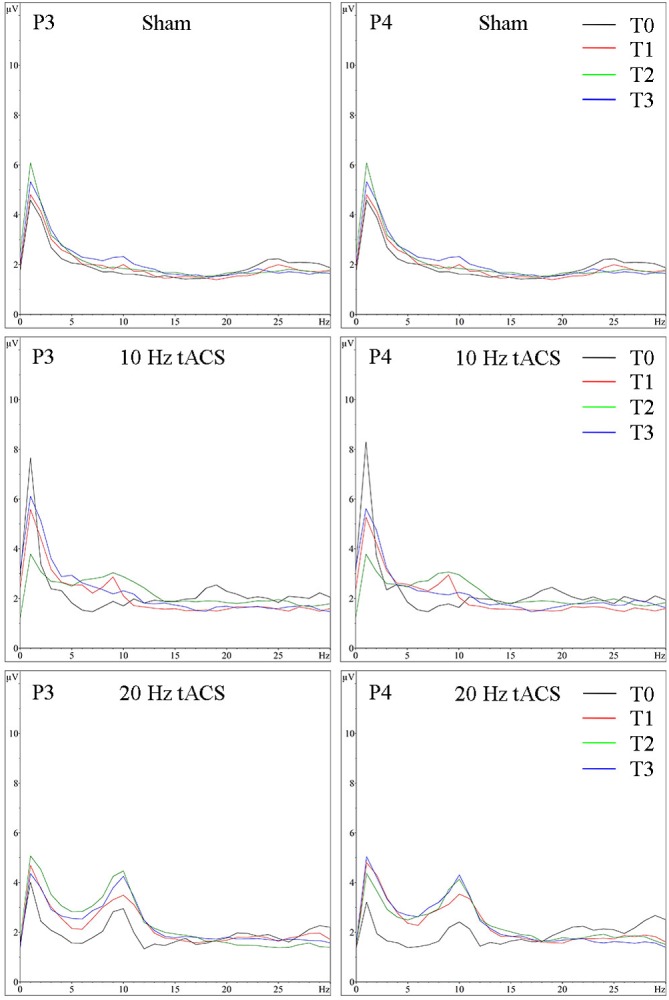
Group averaged EEG activity in rest with eyes open. Frequency spectrum (amplitude in μV) on P3 **(left)** and P4 **(right)** for the 1 min interval with eyes open preceding (T0 = black), immediately after tACS (T1 = red), 30 min after tACS (T2 = green) and 1 day after tACS (T3 = blue) for Sham (at the top), 10 Hz tACS (in the middle), 20 Hz tACS (at the bottom).

For the resting condition with eyes open on P3, a two-way rmANOVA depicted a significant main effect of TIME [*F*(3,63) = 13.294, *p* < 0.01, ${\text{η}}_{\text{p}}^{2}$ = 0.38] indicating that alpha activity is different over time in the stimulated parietal area left hemispheric, independent of GROUP. Additionally, a significant tendency of GROUP [*F*(2,21) = 3.172, *p* = 0.06, ${\text{η}}_{\text{p}}^{2}$ = 0.23] and a GROUP × TIME interaction [*F*(3,63) = 2.765, *p* < 0.05, ${\text{η}}_{\text{p}}^{2}$ = 0.21] emerged, which shows that alpha activity is different over time in the three groups. Bonferroni *post hoc* comparisons clarify a significant increase of alpha activity following 10 Hz tACS up to 30 min (T2–T0; *p* < 0.05) and after 20 Hz tACS up to 1 day after tACS (T3–T0; *p* < 0.05). Furthermore, change in alpha activity from T1 to T0 differs significantly (*p* < 0.05) between 20 Hz tACS and sham controls and from T3 to T0 between 20 Hz tACS and 10 Hz tACS (see **Figure 4C**). For eyes open on P4, a two-way rmANOVA revealed a significant main effect of TIME [*F*(3,63) = 14.135, *p* < 0.01, ${\text{η}}_{\text{p}}^{2}$ = 0.40] indicating that alpha activity is different over time in the stimulated parietal area right hemispheric, independent of GROUP. Additionally, a significant tendency of GROUP [*F*(2,21) = 3.159, *p* = 0.06, ${\text{η}}_{\text{p}}^{2}$ = 0.23] emerged. The GROUP × TIME interaction did not reach significance [*F*(3,63) = 1.550, *p* < 0.17, ${\text{η}}_{\text{p}}^{2}$ = 0.13] (see **Figure 4D**). Frequency spectra of P3 and P4 for the groups Sham, 10 Hz tACS and 20 Hz tACS are presented in **Figure 7**.

### Resting Beta Activity (18–22 Hz) With Eyes Open

**FIGURE 8:**
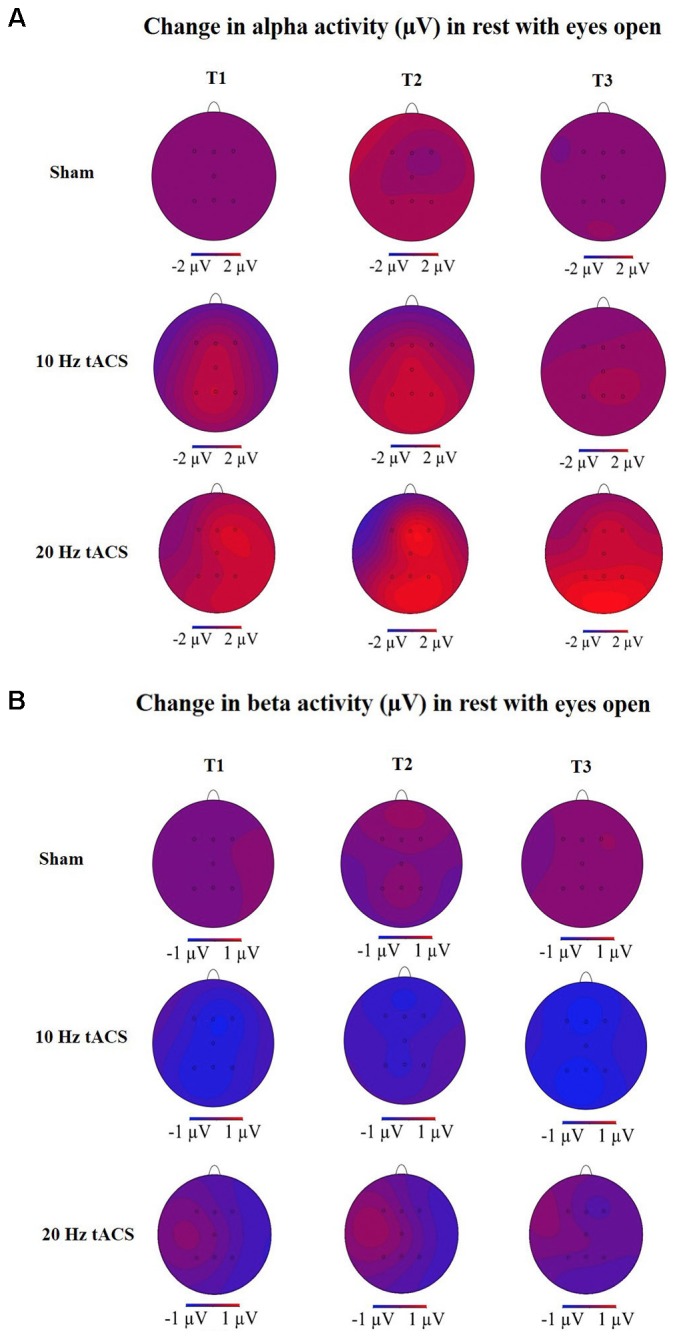
Topographies of group averaged alpha and beta activity changes. **(A)** Changes in alpha activity (μV) in rest from the interval with eyes open preceding tACS (T0) to T1 (immediately after tACS), T2 (30 min after tACS), and T3 (1 day after tACS) for Sham (at the top), 10 Hz tACS (in the middle), 20 Hz tACS (at the bottom). **(B)** Changes of beta activity (μV) in rest from the interval with eyes open preceding tACS to T1 (immediately after tACS), T2 (30 min after tACS), and T3 (1 day after tACS) for Sham (at the top), 10 Hz tACS (in the middle), 20 Hz tACS (at the bottom). Note that these topographies are based on seven electrode positions only.

Statistical analysis of beta-band activity (18–22 Hz) using a three-way rmANOVA with the factors TIME, EEG POSITION, and GROUP revealed neither main effects nor interaction effects. As beta-band was completely unaffected by the stimulation protocol, those values will not be further visually or statistically reported. The topographies for beta activity are presented in **Figure 8B** and depicted that the beta-band was completely unaffected by the stimulation protocol.

### Resting fNIRS Activity (ΔHboxy) With Eyes Open

Relative changes of Hboxy concentration decreased during rest with eyes open, but reached no significance before and after tACS (see Supplementary Tables S4A,B). Additionally, Hboxy *t*-values did not differ from T0 to T1 (see Supplementary Table S4C) indicating that hemodynamic responses during rest with eyes open are not significantly influenced by training or tACS.

### Bimanual Coordination Performance During Task Execution

**FIGURE 9:**
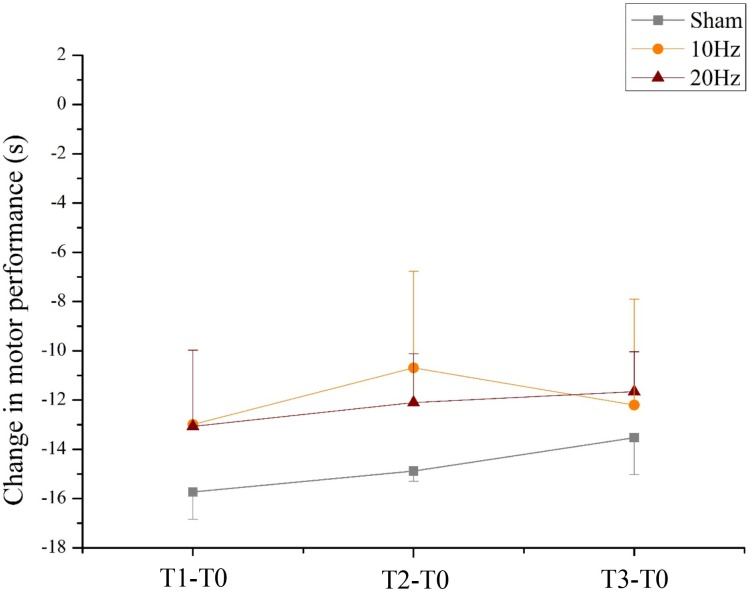
Changes in motor performance. Changes in motor performance (s) from the bimanual coordination task preceding tACS (T0) to T1 (immediately after tACS), T2 (30 min after tACS), and T3 (1 day after tACS) for Sham (gray), 10 Hz tACS (orange) and 20 Hz tACS (ruby). Depicted are means and standard deviations. Note that difference scores are depicted and statistical analysis revealed a significant TIME effect. The main effect is due to significant changes from T0. All statistical details are depicted in the text (see paragraph Bimanual coordination performance during task execution in section “Results”).

For the mean duration of the bimanual coordination task, the two-way rmANOVA showed a statistically significant main effect for TIME [*F*(3,63) = 35.338, *p* < 0.001, ${\text{η}}_{\text{p}}^{2}$ = 0.63]. This result indicates that all participants significantly improved performance in bimanual coordination due to training, independent of tACS. Neither a main effect of group nor a significant TIME × GROUP interaction [*F*(6,63) = 0.405, *p* = 0.087, ${\text{η}}_{\text{p}}^{2}$ = 0.04] was found (see **Figure 9**).

### Alpha Activity (8–12 Hz) During Task Execution

#### Global Effect of tACS

Statistical analysis of alpha activity for all EEG electrodes using a three-way rmANOVA revealed a significant main effect for TIME [*F*(3,63) = 10,701, *p* < 0.01, ${\text{η}}_{\text{p}}^{2}$ = 0.34] and a significant main effect for LOCATION [*F*(3,68) = 3,090, *p* < 0.05, ${\text{η}}_{\text{p}}^{2}$ = 0.13]. The significant main effect for GROUP (*p* < 0.05) was due to a significant difference between the 20 Hz tACS group and the control group. Additionally, both stimulation groups had a significantly increased alpha activity during bimanual coordination; the 10 Hz tACS group up to 30 min, the 20 Hz tACS group up to 1 day after tACS compared to baseline (*p* < 0.05). A significant interaction of TIME x GROUP did not emerge (*p* > 0.05) possibly due to an increasing alpha in the sham stimulated control group. Concerning the statistically significant interaction of TIME × LOCATION [*F*(10,217) = 1,957, *p* < 0.05, ${\text{η}}_{\text{p}}^{2}$ = 0.09], Bonferroni-corrected *post hoc* tests revealed significant differences in alpha activity between P4 and F4 immediately after stimulation and between P4 and Fz 30 min after tACS (*p* < 0.01). These results confirm the location-specific differences following training, and even during motor execution. The different alpha activity evolvement over time for the three groups is visually reflected in the topographies (see **Figure 12A**) by demonstrating stronger alpha activity changes in parietal areas compared to frontal areas.

#### Location-Specific Effect of tACS

**FIGURE 10:**
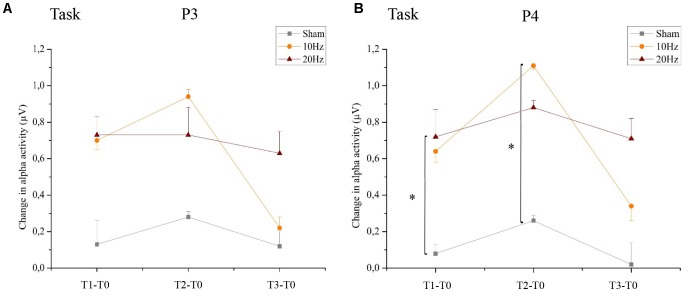
Changes in alpha activity during motor performance. Changes in alpha activity (μV) during the bimanual coordination task preceding tACS (T0) to T1 (immediately after tACS), T2 (30 min after tACS), and T3 (1 day after tACS) for Sham (gray), 10 Hz tACS (orange) and 20 Hz tACS (ruby). Depicted are means and standard deviations. Note that difference scores are depicted and statistical analysis revealed a significant TIME and GROUP^∗^TIME interaction effect. The main effects as well as the interaction effects are due to significant changes from T0. All statistical details are depicted in the text [see paragraph Alpha activity (8–12 Hz) during task execution in section “Results”]. Significantly different changes in alpha activity after tACS to T0 between the groups are marked and depicted with asterisks (^∗^ depicts *p* < 0.05). **(A)** task execution P3, **(B)** task execution P4.

**FIGURE 11:**
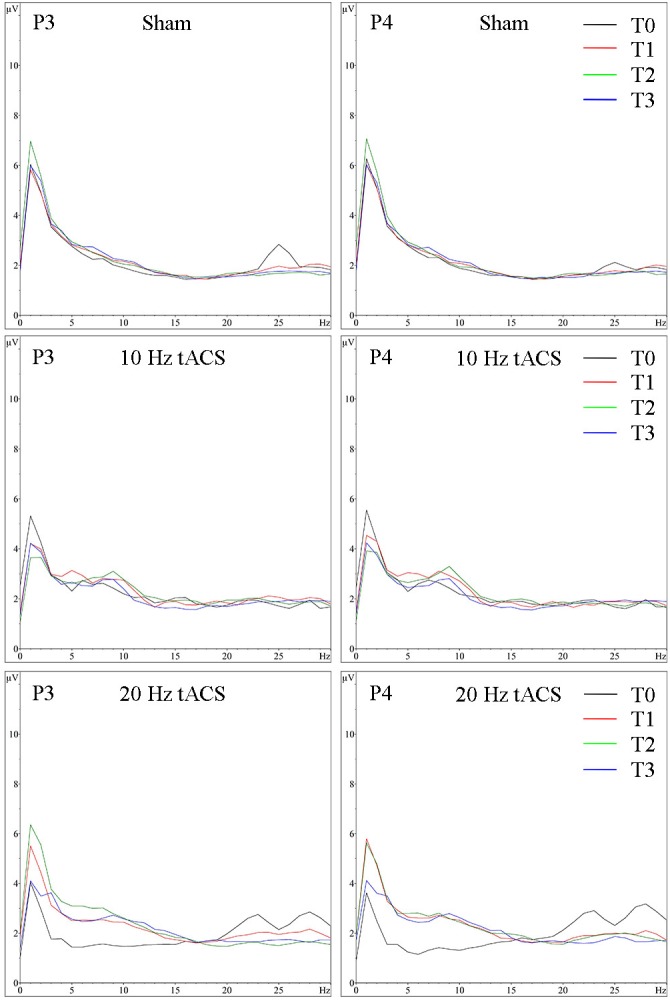
Group averaged EEG activity during motor performance. Frequency spectrum (amplitude in μV) on P3 **(left)** and P4 **(right)** during motor performance in the bimanual coordination task preceding tACS (T0 = black), immediately after tACS (T1 = red), 30 min after tACS (T2 = green) and 1 day after tACS (T3 = blue) for Sham (at the top), 10 Hz tACS (in the middle), 20 Hz tACS (at the bottom).

Analyzing the location-specific effect of tACS on alpha oscillatory activity above P3 during the bimanual coordination task, the two-way rmANOVA revealed a significant main effect for TIME [*F*(3,63) = 9.208, *p* < 0.05, ${\text{η}}_{\text{p}}^{2}$ = 0.31]. Neither main effect GROUP [*F*(2,21) = 1,567, *p* = 0.13, ${\text{η}}_{\text{p}}^{2}$ = 0.09] nor interaction effect of TIME × GROUP [*F*(2,21) = 1,567, *p* = 0.19, ${\text{η}}_{\text{p}}^{2}$ = 0.13] was significant (see **Figure 10A**). In contrast, the two-way rmANOVA for P4 yielded a significant main effect for TIME [*F*(3,63) = 12.987, *p* < 0.01, ${\text{η}}_{\text{p}}^{2}$ = 0.38], a significant main effect for GROUP [*F*(2,21) = 4.400, *p* < 0.05, ${\text{η}}_{\text{p}}^{2}$ = 0.29] as well as a TIME × GROUP interaction [*F*(6,63) = 2.222, *p* < 0.05, ${\text{η}}_{\text{p}}^{2}$ = 0.18]. Thus, alpha activity is different over time in the different groups. Bonferroni *post hoc* analysis exhibited significant differences in alpha activity between 20 Hz tACS and sham controls immediately after tACS compared to T0 (T1–T0) and between 10 Hz tACS and sham controls 30 min after tACS compared to T0 (T2–T0) (see **Figure 10B**). Frequency spectra of P3 and P4 for the groups Sham, 10 Hz tACS and 20 Hz tACS are presented in **Figure 11**.

### Beta-Activity (18–22 Hz) During Task Execution

**FIGURE 12:**
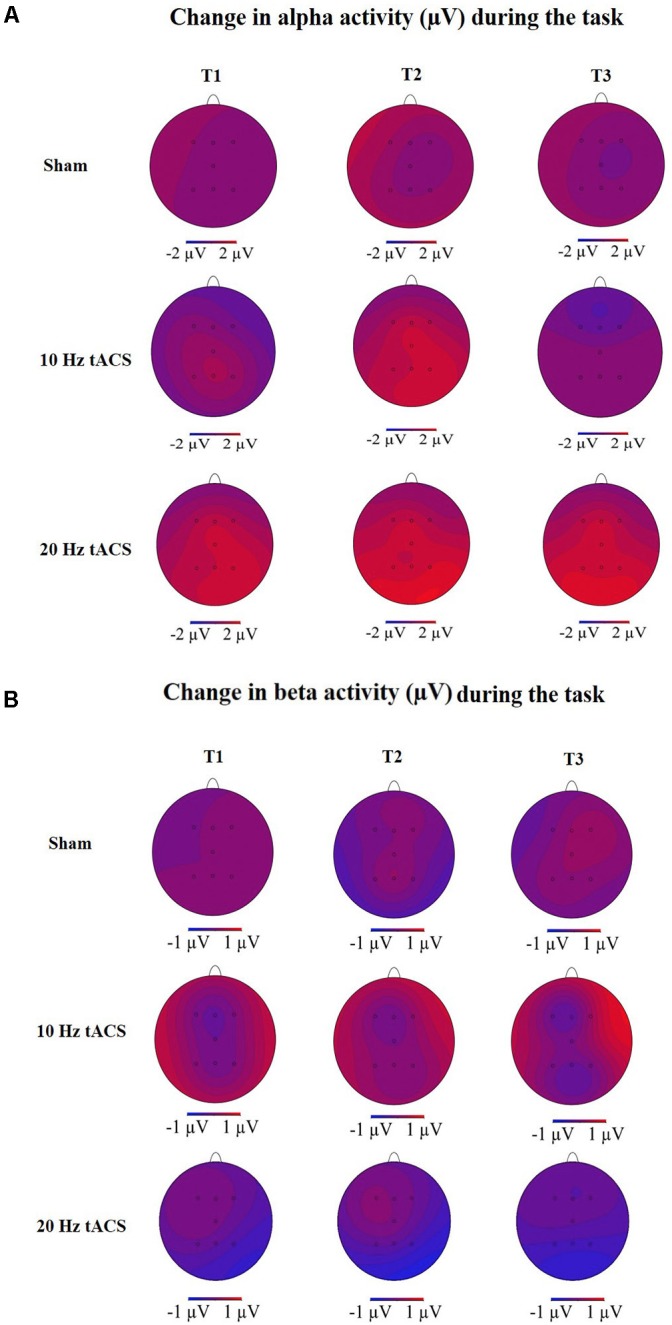
Topographies of group averaged alpha and beta activity changes. **(A)** Changes in alpha activity (μV) during motor performance in the bimanual coordination task preceding tACS (T0) to T1 (immediately after tACS), T2 (30 min after tACS), and T3 (1 day after tACS) for Sham (at the top), 10 Hz tACS (in the middle), 20 Hz tACS (at the bottom). **(B)** Changes in beta activity (μV) during motor performance in the bimanual coordination task preceding tACS to T1 (immediately after tACS), T2 (30 min after tACS), and T3 (1 day after tACS) for Sham (at the top), 10 Hz tACS (in the middle), 20 Hz tACS (at the bottom). Note that these topographies are based on seven electrode positions only.

Statistical analysis of beta-band activity (18–22 Hz) using a three-way rmANOVA with the factors TIME, EEG POSITION, and GROUP revealed neither main effects nor interaction effects. As beta-band was completely unaffected by the stimulation protocol, those values will not be further visually or statistically reported. The topographies for beta activity are presented in **Figure 12B** and demonstrate that the beta-band was completely unaffected by the stimulation protocol.

### fNIRS Activity (ΔHboxy) During Task Execution

**FIGURE 13:**
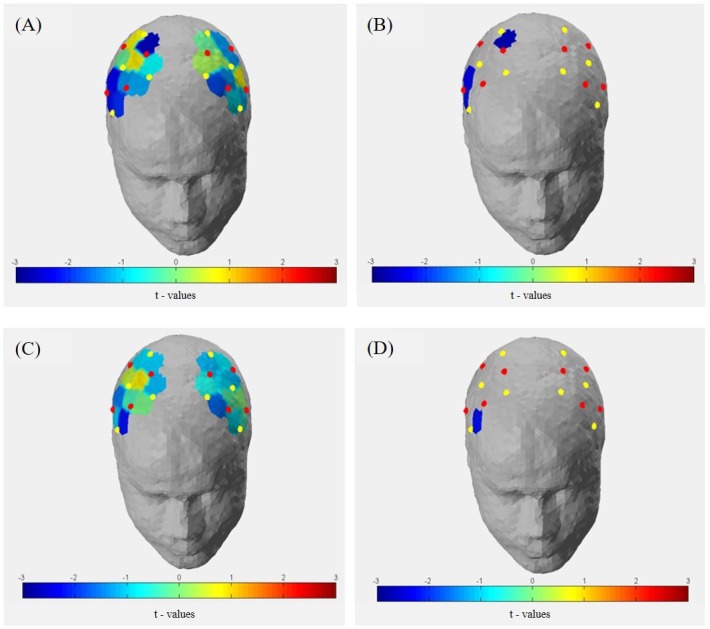
Group averaged t-statistic beta maps. **(A)** t-contrast of 10 Hz tACS vs. Sham for Hboxy concentrations in all channels during the bimanual coordination task at T1. **(B)** Significant channels (*t*-value < –2.4 and >2.4, *p* < 0.05) were depicted colored in the brain map: channel 12 (S-D) covering the primary somatosensory cortex, channel 19 (S-D) covering the primary motor cortex (BA 4) and channel 20 covering the premotor area (BA 6) of the right hemisphere. **(C)** t-contrast of 20 Hz tACS vs. sham for Hboxy concentrations in all channels during the bimanual coordination task at T1. **(D)** Significant channels (*t*-value < –2.4 and >2.4, *p* < 0.05) were depicted colored in the brain map: channel 16 (S6-D8) covering the premotor area (BA 6) of the right hemisphere.

**FIGURE 14:**
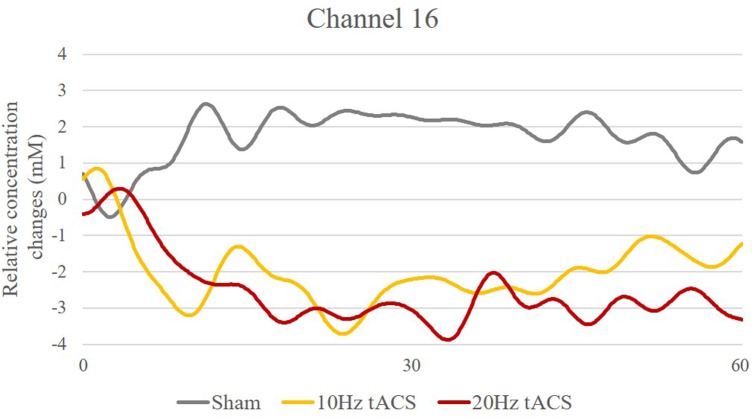
Time-series of Hboxy concentration changes in Channel 16 (Source 6 – Detector 8). Exemplarily, one channel covering the premotor area with significantly different Hboxy concentration time-series for Sham (gray), 10 Hz tACS (orange) and 20 Hz tACS (ruby).

As depicted in **Figure 13**, t-contrasts of the group averaged oxygenated hemoglobin using SPM analysis from the sham control group showed an increase of Hboxy concentration in 17 of 20 channels from T0 to T1. Analysis of ΔHboxy following 10 Hz tACS demonstrated increases in 13 of 20 channels and in 14 of 20 channels following 20 Hz tACS (see Supplementary Table S5). Whereas no group differences were observable at T0, significantly different ΔHboxy concentrations were found between tACS groups and sham controls at T1 (see Supplementary Table S6). During the bimanual coordination task, ΔHboxy concentration decreased significantly in three channels following 10 Hz tACS. These were right hemispheric (Channel 12, Channel 19, and Channel 20) covering regions of the premotor area (BA6), the primary motor cortex (BA4) and the primary somatosensory cortex (BA3/2/1) compared to Sham (see **Figures 2**, **13A,B**). After 20 Hz tACS, one channel of the right hemisphere (Channel 16) covering the premotor area (BA6) revealed a significant decrease in ΔHboxy concentration compared to the control group (see **Figures 2**, **13C,D, 14**). These significant decreases of ΔHboxy concentrations following tACS demonstrate a significant effect of stimulation on the brain’s hemodynamic processes.

### Debriefing

After stimulation, 10 of the 24 participants believed that tACS had a positive effect on motor performance in general. Two participants associated their acute motor enhancement in this study with after-effects of tACS. Beyond that, four of the 24 participants felt certain that they were stimulated by tACS, five participants indicated that they were not stimulated and the others had doubts whether they were stimulated or not. For only five participants, the reported answer is consistent with the applied stimulation suggesting that participants were naïve toward their experimental condition. Results showed no significant relation between suspected and real applied tACS, χ^2^(2) = 4.375, *p* = 0.112, φ = 0.12.

## Discussion

In this study, we combined a complex motor task with simultaneous EEG-fNIRS measures to analyze the after-effects of 10 Hz tACS and 20 Hz tACS on oscillatory activity, hemodynamic changes and bimanual coordination. As expected, high alpha activity during rest with eyes closed was accompanied by decreases in ΔHboxy concentrations, whereby it was not influenced by 10 Hz tACS. During rest with eyes open, alpha activity was significantly increased after 10 Hz tACS and 20 Hz tACS as we have gathered based on the literature (Zaehle et al., 2010) whereas beta band activity stayed unaffected. Furthermore, we hypothesized that parietal 10 Hz tACS evoke synchronized oscillatory activity in the alpha range that lead to reduced interhemispheric interaction and deterioration in bimanual coordination performance (Serrien and Brown, 2002) whereas 20 Hz tACS would promote the natural beta oscillation of bimanual coordination (Rjosk et al., 2016) that leads to an enhanced performance. Contrary to our expectations, parietal alpha activity increased significantly after both stimulation frequencies which was accompanied by significant decreases in ΔHboxy concentrations right hemispheric whereas bimanual motor performance and beta band activity stayed unaffected.

So far, research regarding the effects of tACS focused mostly on either electrophysiological online effects (Helfrich et al., 2014; Neuling et al., 2015; Ruhnau et al., 2016) or electrophysiological after-effects during rest (Zaehle et al., 2010; Neuling et al., 2013; Vossen et al., 2015; Kasten et al., 2016). This is the first study provides insights into tACS induced oscillatory and hemodynamic modulations during a complex motor task using combined electrophysiological and neuroimaging methods.

### tACS Effect on Resting State With Eyes Closed

One of the most interesting findings was that during rest with eyes closed, alpha mean activity significantly increased after application of 20 Hz tACS compared to sham stimulation that persists even until 1 day after stimulation. Whereas Zaehle et al. (2010) or Kasten et al. (2016) demonstrated sustained physiological IAF tACS after-effects (Zaehle et al., 2010) lasting up to 70 min (Kasten et al., 2016), we did not observe any significant after-effects for the 10 Hz tACS on alpha activity. This controversial pattern, however, might be attributed to the performance of the bimanual coordination task concurrently to tACS whereas in the two studies afore-mentioned, participants were at rest during tACS (Zaehle et al., 2010; Kasten et al., 2016). Consequently, brain states during tACS might be one of the decisive factors for the cause of modulatory tACS after-effects on oscillatory brain activity.

Considering the mechanism of entrainment, both 10 Hz tACS and 20 Hz tACS had ratios with the intrinsic frequency that should have principally caused entrainment effects (Herrmann and Strüber, 2017). As a result of frequency-specific after-effects on rest with eyes closed, it is discussible which role the entrainment mechanism has for plastic-related changes evoked through spike-timing dependent plasticity (Feldman, 2012) which are supposed to be the underlying mechanisms for after-effects (Zaehle et al., 2010; Polanía et al., 2012; Vossen et al., 2015). Regarding the hemodynamic changes during rest with eyes closed, ΔHboxy concentrations decreased both before and after tACS. They did not differ significantly which is in line with previous work, where down-regulations of the BOLD signal were observed during rest with eyes closed and which were not modulated by brain stimulation (Vosskuhl et al., 2016).

### tACS Effect on Resting State With Eyes Open

In rest with eyes open, alpha activity significantly increased following 10 Hz tACS and 20 Hz tACS up to 30 min after stimulation compared to Sham. These results confirm previous work from Neuling et al. (2013) who detected significant effects of IAF tACS during rest with eyes open. However, this pattern was not observed in rest with the eyes closed where the natural high amplitude is already too high to be further elevated. This indicates that tACS in the alpha range is more effective when the power of alpha oscillations is lower (Neuling et al., 2013). As opposed to significant changes recently observed in the BOLD signal following 10 Hz tACS (Cabral-Calderin et al., 2016), our fNIRS data do not indicate any significant changes of Hboxy concentrations before and after tACS.

### tACS Effect on Bimanual Coordination Performance

All participants improved their bimanual coordination performance significantly following training. However, immediately after training, the bimanual coordination performance leveled off at a performance of about 20 s for each trial without further improvements across the post measures which indicate a ceiling effect. Moreover, the non-significance of both tACS stimulation effects on bimanual coordination may be attributed to the complexity of the bimanual coordination task. Thus, the frequency-specific effects of 10 Hz tACS and 20 Hz tACS on motor learning (Pogosyan et al., 2009; Pollok et al., 2015; Krause et al., 2016) could not be confirmed due to a possible ceiling effect occurring during training. Pogosyan et al. (2009) demonstrated significant lowered movement times after one session of a bimanual tracking task while participants received concurrently 20 Hz tACS (Pogosyan et al., 2009). As opposed to this, Choe et al. (2016) used both an easy landing task for 10 min and six blocks of 20 trials from the n-back task each day on four consecutive daily sessions. The task performances of both the easy landing task and the n-back task did not change significantly over time possibly due to a ceiling effect (Choe et al., 2016).

### tACS Effect on Neurophysiological Activity During Task Execution

Our electro- and neurophysiological data indicated for the sham group that alpha activity did not increase significantly after training whereas increased ΔHboxy concentrations were observed in most fNIRS-channels covering the motor cortex as it was also reported by Della-Maggiore et al. (2004). This indicates physiological mechanisms underlying training where pre-existing coordination patterns must be suppressed to make space for new patterns (Neva et al., 2012). However, compared to Sham, significantly enhanced alpha activity was observed following 10 Hz tACS and 20 Hz tACS which was accompanied by significant decreases in ΔHboxy concentrations in the right hemisphere. These findings fit well with our expectations, that increased alpha activity is accompanied by decreased hemodynamical activity which is also in line with previous work from Choe et al. (2016) where increased parietal alpha activity correlates with reduced fNIRS beta-values (Choe et al., 2016). Nevertheless, we had hypothesized that 20 Hz tACS does not elicit oscillatory activity in the alpha range which is associated with improved bimanual coordination performance, whereas 10 Hz tACS enhances alpha activity that slows bimanual movements. Contrary to this, the present experiment suggests that 20 Hz tACS enhanced alpha mean activity significantly up to 1 day after stimulation. However, as depicted in **Figure 9**, performance improvements in the bimanual coordination task were lower compared to the improvements of the Sham group, even though they did not achieve significance. Therefore, the interplay between oscillatory activity as well as hemodynamic processes and coordinated bimanual behavior remain an open question since tACS modulate oscillatory activity and hemodynamic changes significantly (Zaehle et al., 2010; Polanía et al., 2012) without significant effects on behavioral outcome. One explanation, however, might be due to the chosen stimulation location: We positioned the HD-tACS electrodes bilaterally on the parietal cortex (P3 and P4) because of its functional role in integrating multi-sensory signals and spatial-temporal coordination of visually controlled movements (Swinnen and Wenderoth, 2004). However, previous studies stimulated the primary motor cortex (M1) and revealed significant effects of tACS on motor learning (Krause et al., 2016; Heise et al., 2017).

Furthermore, based on our data pattern, two difficult and not yet easily answered questions occurred which should be mentioned, and which require further research. On the one hand, we found lateralized effects of tACS. The increased alpha activity between the 20 Hz tACS group and Sham at T1 was significant for the parietal area on the right hemisphere (P4). This was accompanied by significantly decreased Hboxy concentration changes in motor areas also in the right hemisphere. Thus, bilateral induced tACS might evoke changes in oscillatory and hemodynamic activity in intra-hemispheric motor networks. Considering the neural dynamics of hemispheric functions in bimanual coordination, the question arises of how the hemispheric specializations and integrations are organized in bimanual movements? The functional participation of both hemispheres in motor regulation is dynamical and versatile (Serrien et al., 2006). Evidences from callosal patients with bimanual coordination deficits indicate that bimanual patterns rely on interhemispheric couplings (Kennerley et al., 2002) whereby the dominant hemisphere (i.e., the left hemisphere in our population) controls the functional coupling between the motor cortices (Serrien et al., 2003). Both hemispheric asymmetries and an optimal balance between the left and right hemisphere are vital (Serrien et al., 2006). Although the right hemisphere also plays a crucial role for closed-loop aspects of movements dependent on sensory feedback (Haaland and Harrington, 1989) which is indispensable for the realization of goal-directed behavior (Serrien et al., 2006). Various studies highlight the responsibility of the dominant hemisphere for bimanual coordination (Serrien et al., 2003). However, whether this might explain the lateralized physiological effects of tACS without changes in bimanual coordination performance is speculative and requires further research.

On the other hand, we did not find any effects of tACS on brain oscillations in beta frequency. Both stimulation frequencies were applied during bimanual coordination training where entrainment (Antal and Paulus, 2013; Herrmann et al., 2013) and enhanced beta oscillations particularly following 20 Hz tACS were supposed. Whereas beta activity remained unaltered, increased alpha activity evoked by 20 Hz tACS lasted until 1 day after stimulation. One approach might be the ambiguous mechanisms underlying bimanual coordination. Rjosk et al. (2016) demonstrated that beta-band activity plays a crucial role in interhemispheric coordination of movements (Rjosk et al., 2016). Additionally, Davis et al. (2012) suppose that motor control is associated with synchronized oscillatory activity at beta frequency, whereby voluntary movements are associated with suppressed beta band activity (desynchronization) (Davis et al., 2012). Thus, alpha oscillations might have dominated the interhemispheric communication during training which resulted in enhanced alpha band activity during bimanual coordination after both 10 Hz tACS and 20 Hz tACS.

### Limitations and Future Work

The present study’s purpose was to extend the multimodal investigations of tACS effects performed so far by focusing on the modulatory effects of tACS on bimanual coordination and the underlying electro- and neurophysiological mechanisms. This knowledge from a basic research point of view is essential for possible transfers into interventional studies for rehabilitation of motor disorders in patients suffering from neurological diseases. While our results demonstrate a significant modulation of brain oscillatory and hemodynamic activity by tACS, no group differences in bimanual motor learning were observed. Because of the expected learning effect in the bimanual coordination task and the consecutive sessions, no within-subject design was chosen. Future studies should either consider within-subject designs regarding inter-individual variability of tACS effects or they should ascertain the initial skill levels in advance to categorize participants in homogenous groups before prior to alignment. However, the main challenge here and in other studies is the existence of both tACS responders and non-responders with the same tACS protocol due to neuroanatomical and neurophysiological differences on the one side and different tACS effects within an individual over time due to neural plastic changes on the other side. One approach to avoid the “one size fits all” approach could be a closed-loop tACS based on EEG or neuroimaging techniques (Choe et al., 2016). The individualized tACS application may allow for a deeper insight in the mechanisms and effects of tACS which is elementary for future research and the practical transfer although, it will be a big challenge to implement individualized or closed-loop approaches in neurorehabilitation.

Additionally, three other limitations could be mentioned: firstly, the statistical power is relatively low because of *n* = 8 subjects per group. Referring to Choe et al. (2016) where tDCS effects on bimanual motor learning were investigated with EEG and fNIRS in four different groups with comparable sample sizes per group (*n* = 7–10), we focused on the methodological challenge as well. In further studies, the after-effects of tACS could be investigated either at consecutive days or with electrophysiological and neuroimaging methods to consider the complexity and to increase the sample size for higher statistical power. Secondly, no further stimulation conditions in terms of an active control group (e.g., receiving tACS on other brain regions like frontal areas) were included. Thirdly, only two stimulation electrodes were used to rely on previous studies demonstrating tACS effects with larger sponge-electrodes. In future, the eight HD-tACS electrodes could be used to design a montage covering left and right hemispherical motor networks for investigating the causality of altered interregional brain synchronization in patients with bimanual coordination disorders. Additionally, the subject’s baseline performances are crucial for motor skill development and baseline measures of the differing initial skill levels for homogeneous classification were not considered in this study. Thus, subject’s diverse experiences may be relevant in the interpretation of our behavioral findings.

## Conclusion

Based on previous studies which determined either physiological or behavioral effects of tACS, we were able to demonstrate a selective enhancement in (1) brain oscillatory activity in the alpha range and (2) decreases in ΔHboxy concentrations in regions of the premotor area (BA6), the primary motor cortex (BA4) and the primary somatosensory cortex (BA3/2/1) of the right hemisphere following 10 Hz tACS and 20 Hz tACS compared to sham stimulation. The present findings of the simultaneous EEG/fNIRS application represent a valid starting point to close the gap in the tACS literature concerning stimulation effects on bimanual coordination and the underlying electro- and neurophysiological mechanisms. This tACS knowledge is of high importance for basic research and clinical transfer to improve treatments in neurorehabilitation for patients with pathological oscillations which are accompanied by bimanual coordination disorders.

## Ethics Statement

This study was carried out in accordance with the recommen-dations of ‘Ethical Principles of Psychologists and Code of Conduct, APA, Ethikkommission des FB02 Johannes Gutenberg-Universität Mainz’ with written informed consent from all subjects in accordance with the Declaration of Helsinki. The protocol was approved by the ‘Ethikkommission des FB02 Johannes Gutenberg-Universität Mainz’.

## Author Contributions

AB contributed to the conceptualization and realization of the study, performed the data acquisition and the analysis and interpretation, and wrote the manuscript. NP and FS substantially contributed to the data analysis and interpretation and critically revised the manuscript. MD contributed to the conceptualization and design of the study and was involved in the data analysis and interpretation. He critically revised the manuscript, approved the final version and its content, and acted as corresponding author.

## Conflict of Interest Statement

The authors declare that the research was conducted in the absence of any commercial or financial relationships that could be construed as a potential conflict of interest.
