# Supplementary material for: Brain Oscillatory and Hemodynamic Activity in a Bimanual Coordination Task Following Transcranial Alternating Current Stimulation (tACS): A Combined EEG-fNIRS Study
Source: Front Behav Neurosci. 2018 Apr 18;12:67. doi: 10.3389/fnbeh.2018.00067 (PMC5915568; doi:10.3389/fnbeh.2018.00067)
Supplement: Supplementary file 1 [file Table_1.DOCX]

**Supplementary Material: Tables**

**Table 1:** Group averages of Hboxy values for all channels during EC. **(A)** t-contrast at T0, **(B)** t-contrast at T1 **(C)** t-contrast T1 vs. T0

| **(A) T0** | **Ch01** | **Ch02** | **Ch03** | **Ch04** | **Ch05** | **Ch06** | **Ch07** | **Ch08** | **Ch09** | **Ch10** | **Ch11** | **Ch12** | **Ch13** | **Ch14** | **Ch15** | **Ch16** | **Ch17** | **Ch18** | **Ch19** | **Ch20** |
| --- | --- | --- | --- | --- | --- | --- | --- | --- | --- | --- | --- | --- | --- | --- | --- | --- | --- | --- | --- | --- |
| **Sham tACS** | -0,96 | -0,24 | 0,98 | -1,34 | -0,67 | -0,05 | -0,21 | -0,50 | -1,20 | 0,62 | -1,28 | -0,76 | -1,17 | -2,02 | -1,47 | 0,31 | -0,38 | 0,56 | 0,45 | 1,27 |
| **10Hz tACS** | -1,57 | -2,54^*^ | -0,64 | -2,95^*^ | -0,93 | -1,47 | -1,42 | -0,52 | -1,29 | 0,89 | -2,04 | -2,89^*^ | -2,02 | -2,63^*^ | -0,92 | -1,31 | -2,76^*^ | -1,91 | -0,85 | -0,26 |
| **20Hz tACS** | -1,93 | -0,15 | -1,60 | -1,22 | -1,36 | -0,30 | -3,54^*^ | -2,27 | -1,74 | 0,50 | -1,20 | -1,29 | -1,72 | -1,88 | -1,34 | -1,33 | -1,07 | -3,08^*^ | -4,80^*^ | -0,38 |
|  |  |  |  |  |  |  |  |  |  |  |  |  |  |  |  |  |  |  |  |  |
| **(B) T1** | **Ch01** | **Ch02** | **Ch03** | **Ch04** | **Ch05** | **Ch06** | **Ch07** | **Ch08** | **Ch09** | **Ch10** | **Ch11** | **Ch12** | **Ch13** | **Ch14** | **Ch15** | **Ch16** | **Ch17** | **Ch18** | **Ch19** | **Ch20** |
| **Sham tACS** | 0,46 | 0,66 | -0,17 | -0,60 | -1,09 | -0,92 | -0,95 | -0,16 | -0,61 | 0,58 | -1,58 | -0,56 | -1,17 | -1,45 | -0,10 | -0,78 | 1,51 | -0,10 | 0,47 | 0,67 |
| **10Hz tACS** | -2,32 | -0,85 | 0,20 | -1,31 | 1,39 | -1,45 | 0,38 | -1,74 | -1,33 | 0,89 | -2,49^*^ | -1,94 | -2,30 | -2,37 | -1,26 | -1,78 | 0,51 | -1,18 | -1,44 | 0,94 |
| **20Hz tACS** | -1,96 | 0,51 | -1,64 | -1,50 | -2,91^*^ | -3,68^*^ | -2,59^*^ | -1,83 | -3,29^*^ | 0,31 | -1,34 | -1,52 | -1,89 | -2,66^*^ | -2,06 | -0,53 | -0,55 | -2,48^*^ | -2,83^*^ | 0,45 |
|  |  |  |  |  |  |  |  |  |  |  |  |  |  |  |  |  |  |  |  |  |
| **(C) T1-T0** | **Ch01** | **Ch02** | **Ch03** | **Ch04** | **Ch05** | **Ch06** | **Ch07** | **Ch08** | **Ch09** | **Ch10** | **Ch11** | **Ch12** | **Ch13** | **Ch14** | **Ch15** | **Ch16** | **Ch17** | **Ch18** | **Ch19** | **Ch20** |
| **Sham tACS** | 1,04 | 0,67 | -0,84 | 0,11 | -0,81 | -0,64 | -0,74 | 0,05 | -0,28 | -0,07 | 0,85 | 0,01 | -0,03 | 0,04 | 0,43 | -0,84 | 1,55 | -0,48 | 0,23 | -0,12 |
| **10Hz tACS** | -0,35 | -0,11 | 0,57 | 0,82 | 1,65 | -0,09 | 1,30 | -1,05 | 0,03 | -0,44 | -0,32 | -0,22 | -0,12 | 0,27 | -0,51 | -0,32 | 0,84 | 0,07 | -0,27 | 0,85 |
| **20Hz tACS** | -0,14 | 0,52 | 0,03 | -0,88 | -1,07 | -1,72 | 0,11 | -0,55 | 0,94 | 0,02 | -0,04 | 0,13 | 1,17 | -0,92 | -0,44 | 0,22 | -0,48 | 1,11 | 0,58 | 0,59 |

All values presented are in mM concentration units. *****indicates significant t-values (p < .05).
