# Supplementary material for: Brain Oscillatory and Hemodynamic Activity in a Bimanual Coordination Task Following Transcranial Alternating Current Stimulation (tACS): A Combined EEG-fNIRS Study
Source: Front Behav Neurosci. 2018 Apr 18;12:67. doi: 10.3389/fnbeh.2018.00067 (PMC5915568; doi:10.3389/fnbeh.2018.00067)
Supplement: Supplementary file 2 [file Table_2.DOCX]

**Supplementary Material: Tables**

**Table 2:** Hboxy t- values (10Hz tACS vs. Sham and 20Hz tACS vs. Sham) for all channels during eyes closed before tACS (T0).

| **(A) T0** | **Ch01** | **Ch02** | **Ch03** | **Ch04** | **Ch05** | **Ch06** | **Ch07** | **Ch08** | **Ch09** | **Ch10** | **Ch11** | **Ch12** | **Ch13** | **Ch14** | **Ch15** | **Ch16** | **Ch17** | **Ch18** | **Ch19** | **Ch20** |
| --- | --- | --- | --- | --- | --- | --- | --- | --- | --- | --- | --- | --- | --- | --- | --- | --- | --- | --- | --- | --- |
| **10Hz vs. Sham** | -0,18 | -2,09 | -0,93 | -1,51 | -0,88 | -1,46 | -1,39 | -0,35 | -1,11 | 0,84 | 0,99 | -1,47 | 0,92 | -1,41 | -0,80 | -1,22 | -2,15 | -2,12 | -0,96 | -1,12 |
| **20Hz vs. Sham** | -0,91 | 0,01 | -1,74 | -0,77 | -0,94 | -0,12 | -1,97 | -1,74 | -1,33 | 0,29 | 0,86 | -1,06 | 0,47 | 0,43 | -0,39 | -1,22 | -0,71 | -2,52^*^ | -3,24^*^ | -1,31 |

All values presented are in mM concentration units. *****indicates significant t-values (p < .05).


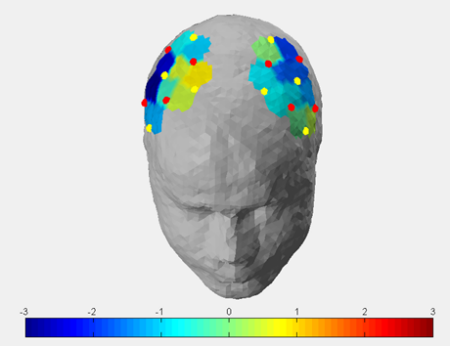

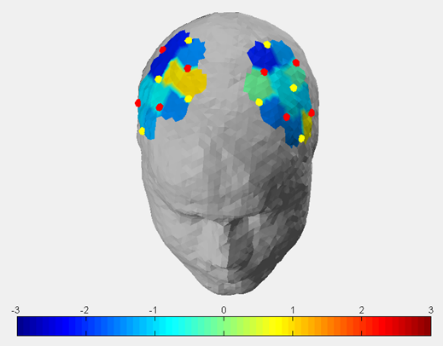


Abb. 2: T-contrast: 20Hz tACS vs. Sham

Abb. 1: T-contrast: 10Hz tACS vs. Sham
