# Supplementary material for: Brain Oscillatory and Hemodynamic Activity in a Bimanual Coordination Task Following Transcranial Alternating Current Stimulation (tACS): A Combined EEG-fNIRS Study
Source: Front Behav Neurosci. 2018 Apr 18;12:67. doi: 10.3389/fnbeh.2018.00067 (PMC5915568; doi:10.3389/fnbeh.2018.00067)
Supplement: Supplementary file 3 [file Table_3.DOCX]

**Supplementary Material: Tables**

**Table 3:** Hboxy t- values (10Hz tACS vs. Sham and 20Hz tACS vs. Sham) for all channels during eyes closed immediately after tACS (T1).

| **Eyes closed T1** | **Ch01** | **Ch02** | **Ch03** | **Ch04** | **Ch05** | **Ch06** | **Ch07** | **Ch08** | **Ch09** | **Ch10** | **Ch11** | **Ch12** | **Ch13** | **Ch14** | **Ch15** | **Ch16** | **Ch17** | **Ch18** | **Ch19** | **Ch20** |
| --- | --- | --- | --- | --- | --- | --- | --- | --- | --- | --- | --- | --- | --- | --- | --- | --- | --- | --- | --- | --- |
| **10Hz vs. Sham** | -1,84 | -0,97 | 0,24 | -0,41 | 1,57 | -1,40 | 0,59 | -1,44 | -1,04 | 0,80 | 0,23 | -1,16 | 0,91 | -1,06 | -1,24 | 0,03 | 0,17 | -1,24 | -1,28 | -0,10 |
| **20Hz vs. Sham** | -1,92 | 0,21 | -1,60 | -1,31 | -0,80 | -0,39 | -0,36 | -1,41 | -0,29 | 0,21 | 0,01 | -1,14 | 0,98 | -0,41 | -0,98 | 0,38 | -0,72 | -1,37 | -1,63 | -0,42 |

All values presented are in mM concentration units. *****indicates significant t-values (p < .05).


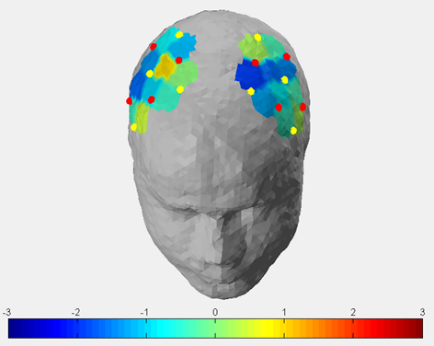

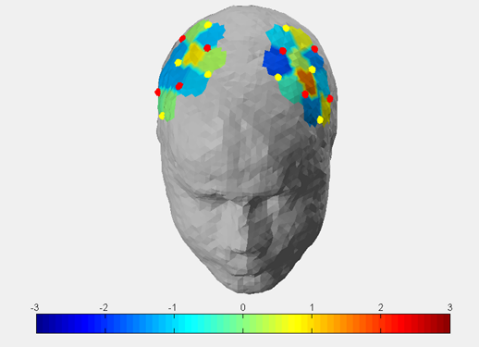


Abb. 4: T-contrast: 20Hz tACS vs. Sham

Abb. 3: T-contrast: 10Hz tACS vs. Sham
