# Supplementary material for: Brain Oscillatory and Hemodynamic Activity in a Bimanual Coordination Task Following Transcranial Alternating Current Stimulation (tACS): A Combined EEG-fNIRS Study
Source: Front Behav Neurosci. 2018 Apr 18;12:67. doi: 10.3389/fnbeh.2018.00067 (PMC5915568; doi:10.3389/fnbeh.2018.00067)
Supplement: Supplementary file 4 [file Table_4.DOCX]

**Supplementary Material: Tables**

**Table 4:** Group averages of Hboxy values for all channels during EO. **(A)** t-contrast at T0, **(B)** t-contrast at T1 **(C)** t-contrast T1 vs. T0

| **(A) T0** | **Ch01** | **Ch02** | **Ch03** | **Ch04** | **Ch05** | **Ch06** | **Ch07** | **Ch08** | **Ch09** | **Ch10** | **Ch11** | **Ch12** | **Ch13** | **Ch14** | **Ch15** | **Ch16** | **Ch17** | **Ch18** | **Ch19** | **Ch20** |
| --- | --- | --- | --- | --- | --- | --- | --- | --- | --- | --- | --- | --- | --- | --- | --- | --- | --- | --- | --- | --- |
| **Sham tACS** | -0,65 | 0,37 | 1,25 | 0,36 | 0,17 | -0,31 | -0,15 | -0,20 | 0,35 | -1,04 | -1,01 | -0,80 | -1,07 | -0,59 | -0,66 | -0,48 | -0,01 | -1,01 | 0,58 | -1,05 |
| **10Hz tACS** | -0,29 | -1,34 | 0,32 | -1,42 | -0,81 | -1,27 | -1,09 | -1,09 | -1,07 | -1,07 | -1,40 | -1,20 | 0,09 | -1,00 | -1,16 | -1,04 | -0,79 | -1,51 | -0,41 | -1,22 |
| **20Hz tACS** | -0,67 | 1,48 | -1,46 | -0,81 | 0,13 | 1,13 | 0,66 | -0,92 | 1,93 | -0,36 | 0,39 | 0,75 | -0,31 | 1,18 | 1,84 | -0,29 | 1,22 | -0,38 | 0,66 | 0,30 |
|  |  |  |  |  |  |  |  |  |  |  |  |  |  |  |  |  |  |  |  |  |
| **(B) T1** | **Ch01** | **Ch02** | **Ch03** | **Ch04** | **Ch05** | **Ch06** | **Ch07** | **Ch08** | **Ch09** | **Ch10** | **Ch11** | **Ch12** | **Ch13** | **Ch14** | **Ch15** | **Ch16** | **Ch17** | **Ch18** | **Ch19** | **Ch20** |
| **Sham tACS** | 0,21 | 1,09 | 0,75 | -0,19 | 2,19 | 0,07 | -0,71 | 1,89 | 1,03 | 1,01 | 1,21 | -0,57 | 0,87 | -1,13 | 0,98 | -1,75 | -1,14 | -0,52 | 2,04 | 0,33 |
| **10Hz tACS** | -1,57 | -1,81 | 0,26 | -0,91 | 0,68 | -0,57 | 0,31 | -1,43 | -1,10 | 0,80 | -0,08 | -1,01 | -1,13 | 0,45 | -1,72 | -1,24 | -1,18 | -0,66 | -1,25 | -1,04 |
| **20Hz tACS** | -1,61 | 0,59 | -1,44 | 1,12 | -0,81 | 0,17 | 0,71 | -1,54 | -0,39 | 0,26 | -0,81 | -0,90 | -0,26 | 0,16 | -1,09 | -1,11 | -0,85 | -1,16 | -1,65 | -0,98 |
|  |  |  |  |  |  |  |  |  |  |  |  |  |  |  |  |  |  |  |  |  |
| **(C) T1-T0** | **Ch01** | **Ch02** | **Ch03** | **Ch04** | **Ch05** | **Ch06** | **Ch07** | **Ch08** | **Ch09** | **Ch10** | **Ch11** | **Ch12** | **Ch13** | **Ch14** | **Ch15** | **Ch16** | **Ch17** | **Ch18** | **Ch19** | **Ch20** |
| **Sham tACS** | 0,61 | 0,64 | -0,06 | -0,35 | 1,79 | 0,24 | -0,42 | 1,60 | 0,77 | 1,41 | 1,49 | 0,03 | 1,38 | -0,93 | 1,09 | -0,80 | -1,10 | 0,74 | 0,65 | 0,88 |
| **10Hz tACS** | -0,76 | 1,07 | -0,06 | -0,31 | 1,02 | 0,37 | 1,14 | -0,72 | 0,00 | 1,17 | 0,99 | -0,08 | -1,05 | 1,07 | 0,04 | -0,76 | -1,01 | 0,50 | -0,44 | 0,68 |
| **20Hz tACS** | -1,16 | -0,55 | 0,87 | 1,15 | -0,79 | -0,93 | 0,18 | -0,04 | -1,26 | 0,44 | -0,89 | -1,03 | 0,22 | -0,78 | -1,99 | -0,81 | -0,89 | -0,84 | -1,72 | -0,93 |

All values presented are in mM concentration units.
