# Supplementary material for: Brain Oscillatory and Hemodynamic Activity in a Bimanual Coordination Task Following Transcranial Alternating Current Stimulation (tACS): A Combined EEG-fNIRS Study
Source: Front Behav Neurosci. 2018 Apr 18;12:67. doi: 10.3389/fnbeh.2018.00067 (PMC5915568; doi:10.3389/fnbeh.2018.00067)
Supplement: Supplementary file 5 [file Table_5.DOCX]

**Supplementary Material: Tables**

**Table 5:** Group averages of Hboxy values for all channels during the bimanual coordination task. **(A)** t-contrast at T0, **(B)** t-contrast at T1 **(C)** t-contrast T1 vs. T0

| **(A) T0** | **Ch01** | **Ch02** | **Ch03** | **Ch04** | **Ch05** | **Ch06** | **Ch07** | **Ch08** | **Ch09** | **Ch10** | **Ch11** | **Ch12** | **Ch13** | **Ch14** | **Ch15** | **Ch16** | **Ch17** | **Ch18** | **Ch19** | **Ch20** |
| --- | --- | --- | --- | --- | --- | --- | --- | --- | --- | --- | --- | --- | --- | --- | --- | --- | --- | --- | --- | --- |
| **Sham tACS** | -0,89 | 0,15 | 0,68 | -1,64 | -0,64 | -1,28 | 0,65 | -0,39 | -1,10 | 0,03 | -1,31 | -0,47 | -1,14 | -1,61 | -1,05 | 0,65 | -0,10 | -1,45 | 0,08 | -0,63 |
| **10Hz tACS** | -1,10 | 0,15 | 0,47 | -2,38^*^ | -1,32 | -1,21 | -1,38 | -1,28 | -1,09 | -1,62 | -1,63 | -2,40^*^ | -1,98 | -4,40^*^ | -1,46 | -1,24 | -1,11 | -0,73 | -1,01 | -0,29 |
| **20Hz tACS** | -1,35 | -1,26 | -1,62 | -1,13 | -2,66^*^ | 0,96 | -2,50^*^ | -2,02 | -1,46 | 0,25 | -1,01 | -1,02 | -0,67 | -1,75 | -1,89 | -1,79 | -0,21 | -1,57 | -2,25 | -0,26 |
|  |  |  |  |  |  |  |  |  |  |  |  |  |  |  |  |  |  |  |  |  |
| **(B) T1** | **Ch01** | **Ch02** | **Ch03** | **Ch04** | **Ch05** | **Ch06** | **Ch07** | **Ch08** | **Ch09** | **Ch10** | **Ch11** | **Ch12** | **Ch13** | **Ch14** | **Ch15** | **Ch16** | **Ch17** | **Ch18** | **Ch19** | **Ch20** |
| **Sham tACS** | -0,62 | 1,59 | 1,34 | 0,81 | 0,21 | -0,49 | 0,73 | 0,87 | 0,24 | 0,23 | 0,90 | 1,67 | -0,98 | 0,35 | -0,56 | 0,68 | -0,46 | -0,85 | 1,36 | 0,78 |
| **10Hz tACS** | -0,36 | 0,05 | 0,72 | -1,66 | -1,37 | -0,95 | -1,16 | -1,02 | 0,87 | -1,13 | 0,71 | -2,08 | -1,51 | -1,27 | -1,48 | -2,40^*^ | 0,77 | -0,64 | -1,98 | -2,42^*^ |
| **20Hz tACS** | -1,02 | -0,92 | -0,82 | -1,54 | -0,13 | -1,02 | -0,49 | -0,95 | 0,28 | -0,21 | -0,88 | -0,96 | -0,26 | 0,30 | -1,13 | -2,20 | -1,02 | -1,80 | -0,94 | -0,57 |
|  |  |  |  |  |  |  |  |  |  |  |  |  |  |  |  |  |  |  |  |  |
| **(C) T1-T0** | **Ch01** | **Ch02** | **Ch03** | **Ch04** | **Ch05** | **Ch06** | **Ch07** | **Ch08** | **Ch09** | **Ch10** | **Ch11** | **Ch12** | **Ch13** | **Ch14** | **Ch15** | **Ch16** | **Ch17** | **Ch18** | **Ch19** | **Ch20** |
| **Sham tACS** | 0,19 | 0,97 | 0,36 | 1,65 | 0,53 | 1,15 | 0,20 | 0,93 | 0,76 | 0,20 | 1,41 | 1,45 | 0,33 | 1,31 | 0,13 | -0,08 | -0,20 | -0,49 | 1,08 | 0,99 |
| **10Hz tACS** | 0,38 | -0,05 | 0,11 | 0,01 | -0,02 | 0,73 | 0,92 | 0,87 | 1,35 | -0,58 | 1,78 | -0,02 | 1,02 | 1,54 | 0,38 | -0,16 | 1,02 | -0,09 | 0,08 | -1,23 |
| **20Hz tACS** | 0,43 | -0,20 | 0,43 | 0,95 | 1,60 | -1,29 | 1,76 | -0,18 | 0,99 | -0,31 | 0,14 | 0,13 | 0,38 | 1,42 | 0,48 | -0,24 | -0,96 | 0,85 | 1,07 | -0,13 |

All values presented are in mM concentration units. *****indicates significant t-values (p < .05).
