# Supplementary material for: Brain Oscillatory and Hemodynamic Activity in a Bimanual Coordination Task Following Transcranial Alternating Current Stimulation (tACS): A Combined EEG-fNIRS Study
Source: Front Behav Neurosci. 2018 Apr 18;12:67. doi: 10.3389/fnbeh.2018.00067 (PMC5915568; doi:10.3389/fnbeh.2018.00067)
Supplement: Supplementary file 6 [file Table_6.DOCX]

**Supplementary Material: Tables**

**Table 6:** Hboxy t- values (10Hz tACS vs. Sham and 20Hz tACS vs. Sham) for all channels during the bimanual coordination task immediately after stimulation (T1).

| **Task T1** | **Ch01** | **Ch02** | **Ch03** | **Ch04** | **Ch05** | **Ch06** | **Ch07** | **Ch08** | **Ch09** | **Ch10** | **Ch11** | **Ch12** | **Ch13** | **Ch14** | **Ch15** | **Ch16** | **Ch17** | **Ch18** | **Ch19** | **Ch20** |
| --- | --- | --- | --- | --- | --- | --- | --- | --- | --- | --- | --- | --- | --- | --- | --- | --- | --- | --- | --- | --- |
| **10Hz vs. Sham** | 0,26 | -0,18 | 0,25 | -1,79 | -1,39 | -0,93 | -1,36 | -1,34 | 0,78 | -1,14 | -0,83 | -2,64^*^ | 0,89 | -1,24 | -1,32 | -2,04 | 0,83 | -0,13 | -2,34^*^ | -2,19^*^ |
| **20Hz vs. Sham** | -0,65 | -1,19 | -1,01 | -1,67 | -0,24 | -0,92 | -0,86 | -1,16 | 0,01 | -0,28 | -1,20 | -1,04 | 0,91 | -0,08 | -0,33 | -2,16^*^ | -1,00 | 0,40 | -1,53 | -0,98 |

All values presented are in mM concentration units. *****indicates significant t-values (p < .05).
